# End-to-end automated microfluidic platform for synthetic biology: from design to functional analysis

**DOI:** 10.1186/s13036-016-0024-5

**Published:** 2016-02-02

**Authors:** Gregory Linshiz, Erik Jensen, Nina Stawski, Changhao Bi, Nick Elsbree, Hong Jiao, Jungkyu Kim, Richard Mathies, Jay D. Keasling, Nathan J. Hillson

**Affiliations:** Fuels Synthesis and Technologies Divisions, Joint BioEnergy Institute, Emeryville, CA 94608 USA; Biological Systems and Engineering Division, Lawrence Berkeley National Lab, Berkeley, CA 94720 USA; DNA Synthesis Science Program, DOE Joint Genome Institute, Walnut Creek, CA 94598 USA; Chemistry Department, University of California, Berkeley, CA 94720 USA; HJ Science & Technology Inc., Berkeley, CA 94710 USA; Present address: Tianjin Institute of Biotechnology, Chinese Academy of Sciences, Tianjin, China; Present address: Department of Mechanical Engineering, Texas Tech University, Lubbock, TX 79409 USA; Department of Chemical & Biomolecular Engineering and Department of Bioengineering, University of California, Berkeley, CA 94720 USA

**Keywords:** Synthetic biology, Microfluidics, DNA assembly, Transformation, Cell culture, Analysis

## Abstract

**Background:**

Synthetic biology aims to engineer biological systems for desired behaviors. The construction of these systems can be complex, often requiring genetic reprogramming, extensive de novo DNA synthesis, and functional screening.

**Results:**

Herein, we present a programmable, multipurpose microfluidic platform and associated software and apply the platform to major steps of the synthetic biology research cycle: design, construction, testing, and analysis. We show the platform’s capabilities for multiple automated DNA assembly methods, including a new method for Isothermal Hierarchical DNA Construction, and for *Escherichia coli* and *Saccharomyces cerevisiae* transformation. The platform enables the automated control of cellular growth, gene expression induction, and proteogenic and metabolic output analysis.

**Conclusions:**

Taken together, we demonstrate the microfluidic platform’s potential to provide end-to-end solutions for synthetic biology research, from design to functional analysis.

**Electronic supplementary material:**

The online version of this article (doi:10.1186/s13036-016-0024-5) contains supplementary material, which is available to authorized users.

## Background

Synthetic biology currently relies heavily on trial and error, and debugging and reprogramming complicated biological systems continues to require significant resources [1]. While recent efforts have importantly established some physical and informatics standards for synthetic biology [2], the time required to reach a desired behavior remains very lengthy. Furthermore, the high-throughput generation of reliable and reproducible experimental data is still challenging and requires extensive laboratory automation [3, 4].

**Fig. 1:**
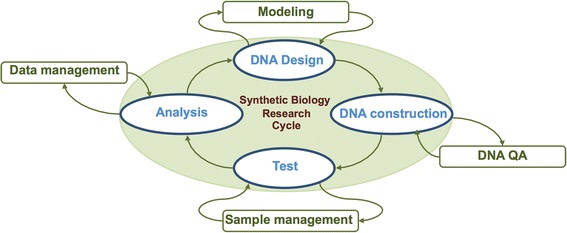
Synthetic biology research cycle for the development of new biological systems

The synthetic biology research cycle, integrating design, construction, testing, and analysis (Fig. 1), is a systems-development approach shared in common with many engineering disciplines [5]. The adoption of the integrated development cycle as a standard approach in synthetic biology could significantly reduce the time to product, improve the likelihood of producing desired functionalities, and make the development of biological systems fast, inexpensive, and robust [3].

Biology-friendly automated platforms and software tools are crucial for modernizing the life sciences. While liquid-handling robotics can accelerate research and provide efficient solutions, they remain expensive, have large footprints, and require large sample volumes (which can be prohibitively expensive for high-throughput experiments). Performing laboratory operations in small volumes and increasing throughput using miniaturized microfluidic Lab-on-a-Chip (LOC) devices is the next step forward in biotechnology [6, 7]. For synthetic biology research automation in particular, a universal and programmable microfluidic sample-processing platform, capable of performing a broad range of operations and integrating and automating the major steps of the development process, is required.

A great variety of microfluidic devices have been demonstrated for sample processing applications [8]. However, the majority of these devices is limited to performing specific tasks, and therefore has not achieved integrated, end-to-end synthetic biology automation. A recently reported hybrid digital/droplet microfluidic device [9] approaches to this end-to-end integrated automation, but starts with DNA assembly (post design and DNA fragment preparation) and stops after transformation (before testing and analysis). The multipurpose microfluidic platform described herein uses pneumatically actuated microvalve technology, enabling a wide range of miniaturized sample processing operations with precise metering and mixing capabilities, simplified scaling-down of experiment protocols, and minimized reagent dead volumes [10, 11].

2D microvalve array technology has been used as a programmable sample processing architecture for numerous chemical and biological analysis procedures [10, 12–14]. Digital transfer of fluids within a 2D array enables precise and rapid reagent routing, mixing, rinsing, serial dilution, and storage/retrieval operations. Furthermore, this technology allows rapid processing of sample volumes ranging from the nanoliter to microliter scale. The programmability, precision, and robustness of this technology are ideally suited for the implementation of a diverse set of synthetic biology applications.

## Results and Discussion

### Platform components

Our automated multipurpose platform consists of a microfluidic chip (implemented using 2D microvalve array technology), an electronic pneumatic control system [15], a temperature regulation system, and computational software. The microfluidic platform features 150 nL transfer precision of each as well as the ability to do multiple transfers to yield microliter volumes. The platform is managed by PR-PR [16, 17], a high-level programming language for laboratory automation with a web-based interface, which translates user-defined sample processing operations into a sequence of commands for microvalve control at the machine level. PR-PR output is processed by LabView software (National Instruments), which transmits the operational commands to an array of miniature solenoid valves. Each solenoid valve switches between positive pressure (closing) and vacuum (opening) states, and controls a single microvalve within the 2D array (Additional file 1: Figures S1 and S2). A number of approaches for multiplex addressing of valves that reduce the number of solenoids per valve have been previously reported [15]. Liquids can be transported on-chip by a programmable actuation of sequences of microvalves in a peristaltic fashion.

**Fig. 2:**
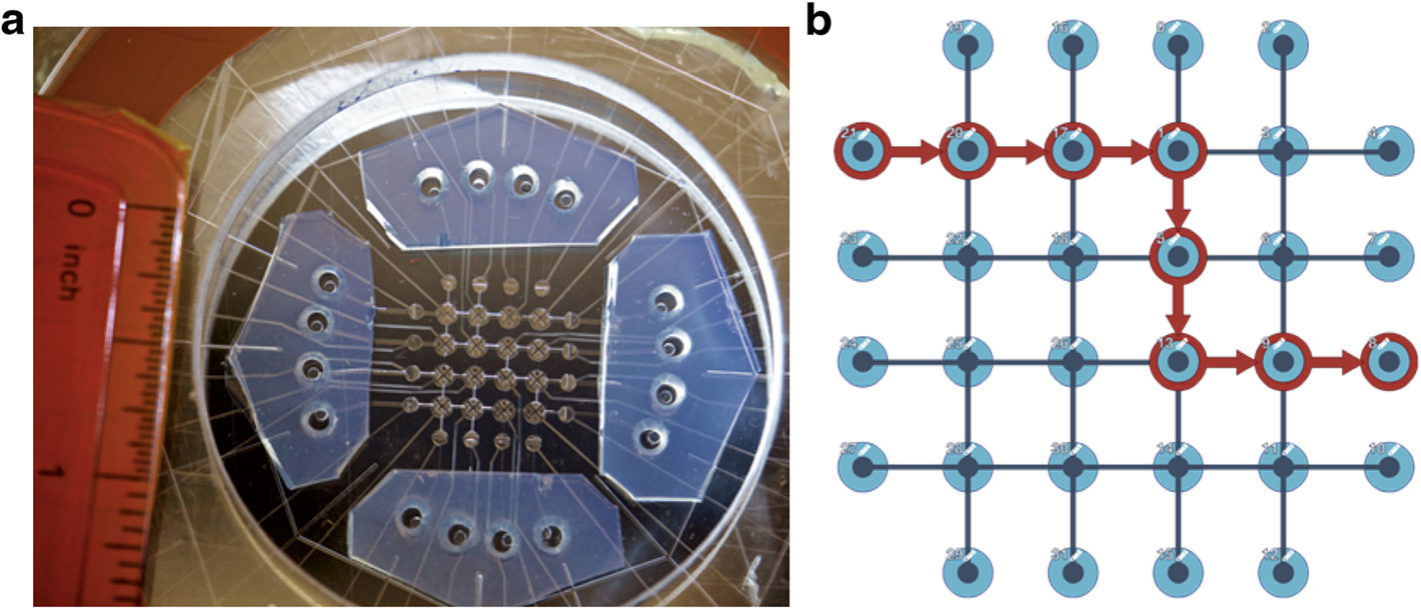
Microfluidics chip. **a** Photograph of the physical microfluidics chip (ruler for scale). 16 input/output macro scale wells (~20 uL working volume; blue-colored regions) surround internal micro scale valves (~150 nL). **b** PR-PR software user-interface schematic representation of the microfluidics chip. The red arrows show a representative example of a reagent transfer path through the chip from macro scale input well [21] to macro scale output well [8], through internal micro scale values [1, 5, 9, 13, 17, 20]

Since many laboratory procedure steps can be described as series of material transfers, even very complex laboratory protocols can often be presented as sequences of transfer commands implemented in PR-PR. Each PR-PR transfer statement consists of four elements: Source, Destination, Amount, and Method. The same four elements are applicable to liquid transfer across all platforms supported by PR-PR. We consider the microfluidic chip as undirected graph Graph = (Vertices, Edges), where valve junctions and input/output wells represent vertices (nodes) and connecting channels represent edges. A graph search algorithm is implemented for finding the most efficient path through the chip, between the source and the destination locations (Fig. 2). It is possible to assign reagents used in a protocol to specific locations, which permits a high level of protocol abstraction and enables users to refer to a particular location by the corresponding reagent name. PR-PR also allows configuring parameters of laboratory workspace, such as microfluidic device topology, robotic worktable etc. through its biology-friendly Graphical User Interface (GUI).

### Process steps

Our microfluidic platform integrates and automates the key steps of the iterative synthetic biology design-construct-test-analyze research cycle [18] (Fig. 1), composed of: 1) Design of DNA libraries performed by ‘DNA constructor’ software and design of construction protocols by PR-PR [16]. 2) Construction: DNA synthesis and transformation into different hosts. In particular, we have automated various methods of DNA synthesis, such as Gibson [19] and Golden Gate assembly [20] and the Isothermal Hierarchical DNA Construction (IHDC), which is our novel method of de novo DNA assembly, especially developed for the microfluidics environment. Further, we implemented the transformation of the constructed DNA molecules into two distinct hosts, *E. coli* and *S. cerevisiae*. 3) Test: we performed on-chip functional assays, including cell growth, protein expression induction, and colorimetric assay; 4) Analysis: we performed image analysis of on-chip automated experiments for evaluation of desired function. Below, we describe each of these process steps in greater detail.

### Design

#### DNA constructor

Software supporting the design of complex combinatorial DNA libraries and the optimization of their corresponding DNA construction protocols is critical to the efficient creation of new biological systems. We have developed ‘DNA Constructor’, a web-based application which designs optimized hierarchical construction protocols for large DNA molecules (Additional file 1: Figures S3a and S4a) and combinatorial DNA libraries (Additional file 1: Figure S5). DNA Constructor allows users to specify the desired DNA library (via the DNA Constructor scripting language Additional file 1: Figure S6) and parameters (e.g., maximum primer length) for customizing the protocol generation algorithm. The generated hierarchical construction protocols minimize nonspecific products and are optimized to achieve the construction in the fewest number of steps. This allows efficient construction of long DNA molecules and combinatorial DNA libraries by re-using components shared between variants and incorporation of available existing DNA fragments.

### Construction

#### Isothermal Hierarchical DNA Construction (IHDC)

**Fig. 3:**
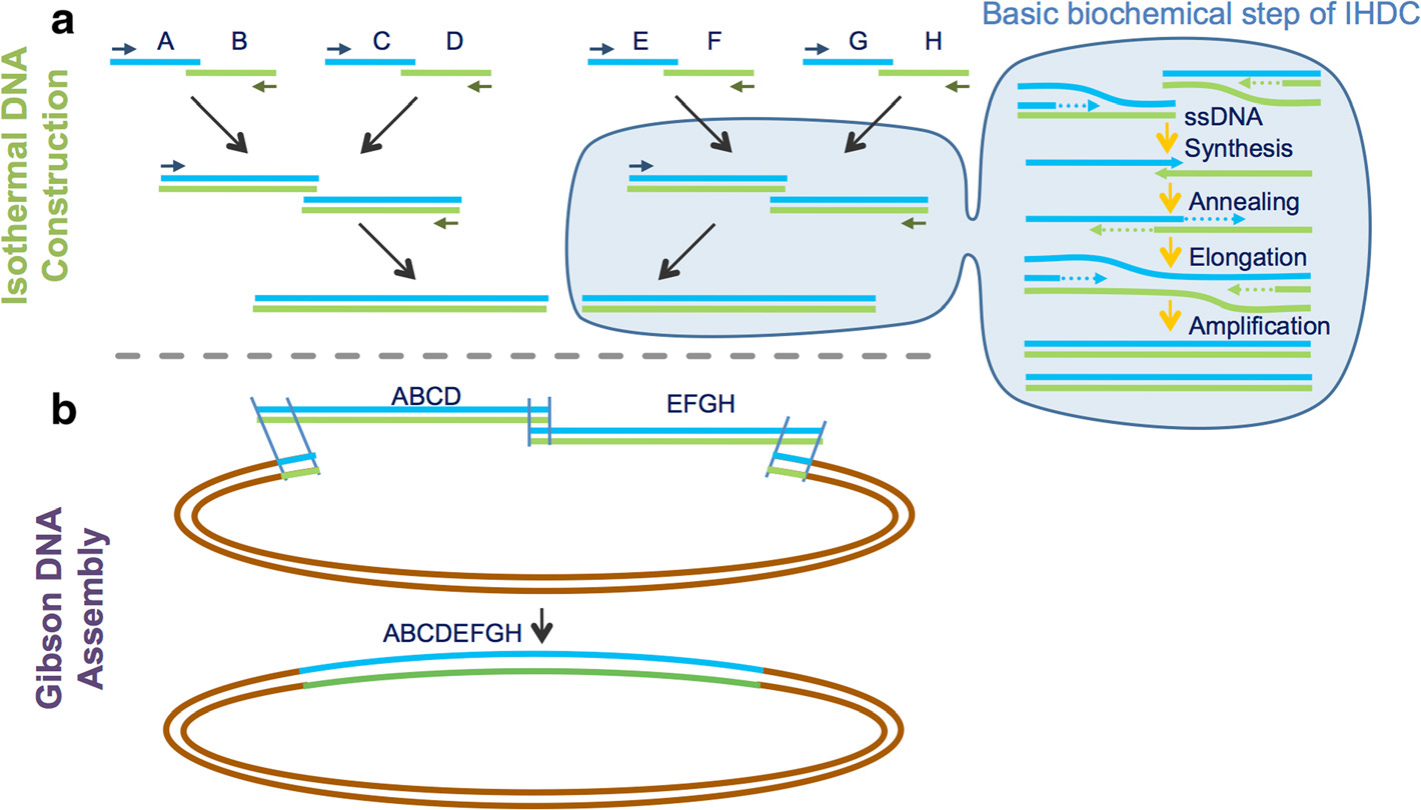
Isothermal hierarchical DNA construction (IHDC) coupled with Gibson DNA assembly. **a** Isothermal hierarchical DNA construction. At left, an example of a three-level hierarchical construction tree starting with eight oligonucleotides. At right, schematic description of the basic biochemical step of IHDC. **b** Gibson DNA assembly. Integration of IHDC-constructed fragments with Gibson DNA assembly

We developed a new method for hierarchical DNA construction in isothermal conditions, especially optimized for implementation on our microfluidics platform. IHDC offers significant advantages over PCR-based methods including reduction of control equipment, faster processing times, and better amenability to direct automation with microfluidic technology. Isothermal DNA construction takes as an input two overlapping dsDNA and produces an elongated dsDNA as output. Primers are recombinase-incorporated between the strands, and then polymerase-elongated to produce ssDNA. The overlapping ssDNA molecules hybridize, priming each other for an overlap extension elongation reaction to form dsDNA, which is then amplified by isothermal amplification to yield the desired elongated dsDNA (Fig. 3). The mechanism of IHDC is derived from Recombinase Polymerase Amplification (RPA) [21]. RPA is an isothermal DNA amplification process that is capable of pairing primers with homologous sequences in duplex DNA. We modified the reaction conditions and adapted them to the IHDC method.

**Fig. 4:**
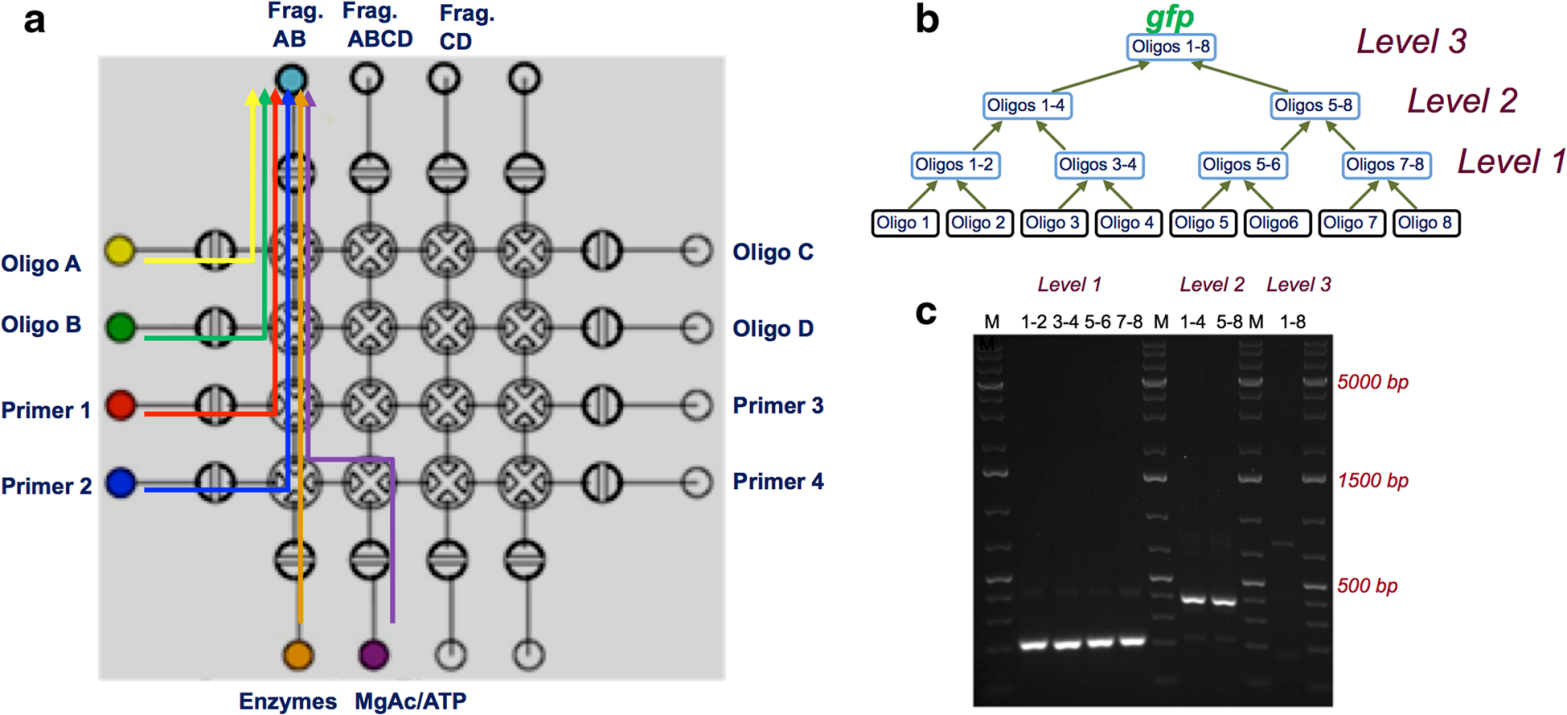
Overview of isothermal DNA construction on the microfluidics platform. **a** Overview of the basic IHDC step on the microfluidics platform. Stage I. Two oligos A and B (as shown, or alternatively two DNA fragments A and B) and a mixture of enzymes are transferred to the reactor. Stage II. Primers P1 and P2, and a mixture of enzymes, are transferred to the reactor. Stage III. A mixture of ATP and magnesium acetate, and a mixture of enzymes, are transferred to the reactor. The temperature is increased to 38 °C, and the reaction is incubated for 15 min. As a result, an elongated and amplified DNA fragment AB is produced. **b** Hierarchical construction tree of seven separate synthesis reactions that result in the final product (gfp as shown). **c** Gel electrophoresis image of all the intermediates and the final gfp construct. Lanes as labelled by: M: GeneRuler 1 kb Plus DNA Ladder (Thermo Scientific); Level 1 (quarter) fragments: 1-2, 3-4, 5-6, 7-8; Level 2 (half) fragments: 1-4 and 5-8; Level 3 (full length gfp) fragment: 1-8

We have demonstrated automated Isothermal Hierarchical DNA Construction on our microfluidic platform. Figure 4a shows a schematic of the automated IHDC reaction protocol on the microfluidic chip. This assembly protocol was used to build a 754 bp construct from eight synthetic oligos. Figure 4b shows the hierarchical construction tree consisting of seven separate synthesis reactions to form the final product. Particularly, on the current microfluidic chip that has 16 input/output wells we have performed two hierarchical steps in a fully automated manner, resulting in DNA molecules that encode the entire RFP (Additional file 1: Figure S4) and two halves of GFP. For construction of the whole GFP-encoding DNA molecule, given the lack of sufficient input/output wells on the current chip, we have reloaded the chip with the two previously constructed halves along with two primers and performed an additional IHDC step. Between reloading the reagents, the valves were washed with an automated rinsing program. The assembly times for GFP and RFP were less than two hours and one hour, respectively, with each individual IHDC step requiring only 15 min for completion. Figure 4c shows gel electrophoresis image of all the intermediates and the final GFP construct. The results of RFP construction are shown in Additional file 1: Figure S4b. These results indicate high yields of intermediate and final products of the correct length. Our automated DNA construction method is therefore highly effective and can be scaled to larger assembly lengths and combinatorial sets.

#### Gibson assembly

Using our automated microfluidic platform, synthetic DNA fragments generated by IHDC were integrated with expression vector pETBlue-1 by Gibson assembly [19]. Gibson assembly allows joining multiple DNA fragments in a single, isothermal reaction. We have adapted the Gibson method to our microfluidic platform and integrated our IHDC method with Gibson assembly. The output DNA fragments of IHDC were designed to be compatible with the Gibson method. In particular, to insert full GFP or RFP coding sequences into the pETBlue-1 plasmid, digested by EcoRV on the microfluidic platform (Additional file 1: Figure S7), we created overlapping regions during the IHDC stage by addition of the regions surrounding the EcoRV restriction site on the pETBlue-1 plasmid to the GFP or RFP DNA molecule sequences. Alternatively, when inserting the GFP as two fragments created by the IHDC method into the plasmid, the overlapping region for Gibson assembly was designed in the middle of the GFP DNA sequence (Additional file 1: Figure S8a). Each DNA fragment constructed by IHDC and the pETBlue-1 linearized plasmid were purified by QIAquick PCR Purification Kit (Qiagen) and reloaded into the microfluidic chip. The Gibson DNA assembly was automated by running the PR-PR output script. The scheme of the automated Gibson assembly is shown in Additional file 1: Figure S8b. After the assembly, the reactions were incubated at 50 °C for 30 min on the chip. As a result, the chip produced circularized, ready for transformation, pETBlue-GFP (Additional file 1: Figure S8b) and pETBlue-RFP plasmids containing de novo synthesized GFP and RFP DNA fragments. These plasmids were used in automated E. coli transformations described below. In our previous work, we have also demonstrated on-chip hierarchical Gibson assembly of up to eight DNA fragments yielding a 12 kbp plasmid [16].

#### Transformation of *E. coli*

Using our microfluidic platform we automated the transformation of the newly assembled pETBlue-GFP and pETBlue-RFP plasmids into the *E. coli* host strain Tuner (DE3) pLacI (Novagen). The chemically competent *E. coli* cells and the Gibson assembly mixture (i.e., assembled pETBlue-GFP or pETBlue-RFP plasmids) were loaded into the microfluidic chip cooled to 0 °C using an external Peltier temperature controller. The DNA plasmids were transferred to the wells containing the competent *E. coli* cells (Additional file 1: Figure S9). The DNA and the cells were incubated for 10 min at 0 °C and then the heat shock was performed at 42 °C for 45 s. Then the transformation mixture was cooled to room temperature and the cells were incubated with SOC medium for half an hour at 37 °C. Ultimately all the cells were plated on LB-Amp agar plates and incubated at 37 °C over night to produce transformed *E. coli* colonies containing the desired plasmids.

#### Golden Gate assembly of combinatorial library

We have previously demonstrated the capability of the microfluidic platform for the assembly of combinatorial DNA libraries by constructing a library of 16 variants by the Golden Gate assembly method [22]. Design of the library was done using j5 [23] and Device Editor [24]. All variants shared the same backbone p4001 and combination of one promoter variant with one variant of bicistronic design (BCD) coupled with GFP gene (Additional file 1: Figure S10a). The architecture of the current microfluidic chip allows assembly of up to 8 variants in parallel, therefore to produce a 16-variant library, the experiment was run twice. Schematics of reagents and reaction allocations are presented in Additional file 1: Figure S10b. After assembly, the reactions were incubated at room temperature and transformed into *E. coli* cells. After the transformation and induction of expression of GFP by addition of IPTG, we saw different levels of GFP expression for different variants. We verified the quality of all the 16 constructed library variants by PCR colony screening and sequencing [16].

#### DNA assembly in yeast

**Fig. 5:**
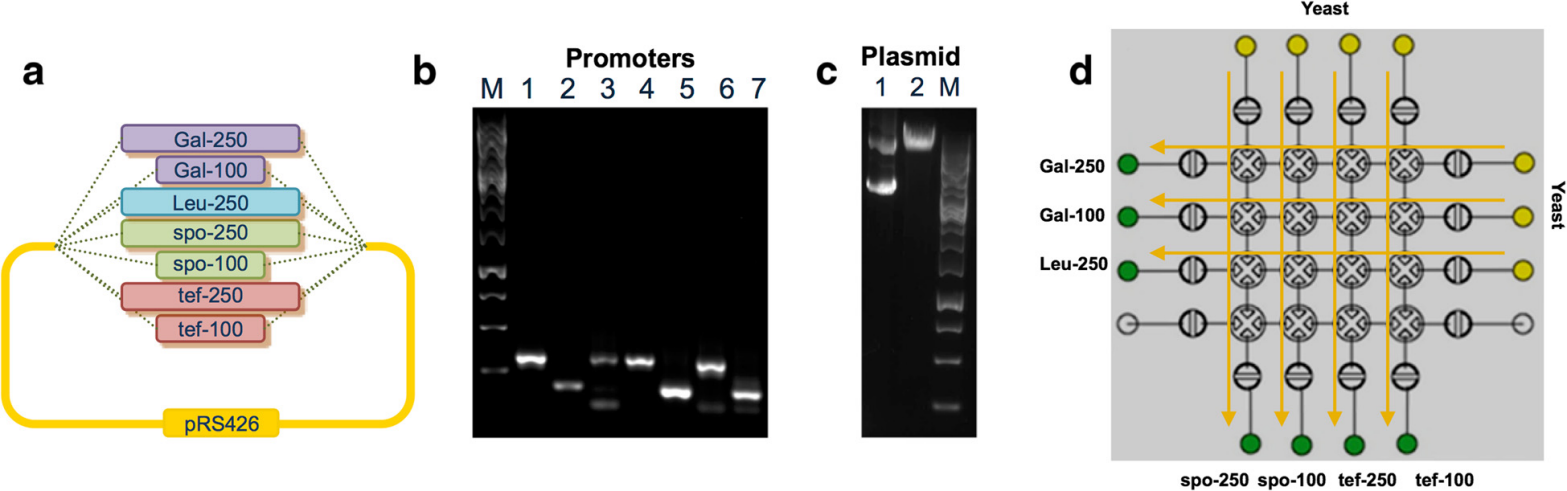
Construction in yeast of a promoter library. **a** Promoter library schematic. **b** Gel electrophoresis image of amplified promoters: lane 1, Gal-250; lane 2, Gal-100; lane 3, Leu-250; lane 4, spo-250; lane 5, spo-100; lane 6, tef-250; lane 7, tef-100. **c** Gel electrophoresis image of: lane 1, pRS426; lane 2, pRS426-yeGFP EagI digest. M is GeneRuler 1 kb Plus DNA Ladder (Thermo Scientific). **d** Schematic of reagent transfers through the microfluidic chip. The green input wells contain a mixture of different promoters and digested plasmid pRS426 with Salomon sperm DNA as a carrier. The yellow input wells contain *S. cerevisiae* competent cells. Arrows show pathways of reagent transfer on-chip according to the automated protocol

To demonstrate automated transformation and in vivo DNA assembly in yeast on the microfluidic platform we have created a library of seven constructs with different promoters that control expression of GFP. To isolate the seven promoters we amplified two variants of 250 bp and 100 bp of the following four promoters: gal1, leu2, spo13, tef1 from *S. cerevisiae* genomic DNA (Fig. 5). As a backbone for in vivo DNA construction we used plasmid pRS426-yeGFP, which was linearized on-chip by EagI restriction enzyme digestion. After restriction, the purified plasmid and each promoter amplicon separately were mixed with Salmon sperm DNA (Clontech) as a carrier. The yeast cells were grown overnight, washed twice in water and re-suspended in PEG and lithium acetate solution. The DNA mixture and cell solution were loaded into separate input wells on-chip. The *S. cerevisiae* competent cells were automatically transferred to wells with preloaded DNA mixture according to the PR-PR protocol and the chip was incubated for two hours at 42 °C. Then, the *S. cerevisiae* cells were plated onto rich, solid medium lacking uracil. After the overnight incubation, yeast colonies with various levels of green fluorescence were observed as a result of the various levels of GFP expressed from the different promoters (Additional file 1: Figure S11).

### Test and evaluation

Screening assays are substantially important for the development of new biological systems. Such assays are required to validate the function of the engineered system and quantify production levels of desired products. In the present study, we demonstrated the capability of our microfluidic platform to perform functional assays through automated gene expression induction, phenotype screening, and isopentenol measurement. These functional assessments are not necessarily more high-throughput than alternative methods, but the device described here enables a more streamlined assessment process and reduced instrumentation and reagents costs (see Additional file 1 for time and cost points of comparison with conventional laboratory automation systems).

Protein expression induction and phenotype screening assay. We have evaluated on our microfluidic platform the expression of the de novo constructed fluorescent protein GFP. After transformation, 18 random clones, containing pETBlue-GFP construct, were plated on LB-Amp-IPTG agar plates. The plates were incubated overnight. Seven clones containing error-free GFP constructs developed green color (Additional file 1: Figure S12). One of the colonies of green color phenotype was incubated in LB medium with Ampicillin in twelve wells on the chip. We loaded IPTG in two wells and transferred it into six wells containing cells. After incubation at room temperature with 1 mM IPTG for 8 h, we took an image of the chip using a transilluminator and a CCD camera with filter (Additional file 1: Figure S13). We performed image analysis by measurement of average intensity over squares of size 21x21 pixels with centers in wells (green circles) and then calculated the relative fluorescent intensity of induced and non-induced wells. Based on the relative fluorescent intensity, GFP expression level of the induced cells was 8.6 times higher than the non-induced cells. The results of our on-chip evaluation clearly show that the cells containing de novo synthesized DNA molecules encoding gfp, express GFP protein after induction by IPTG.

#### MBTH assay

Isopentenol is an excellent alternative to fossil fuels [25]. However, it is not widely produced by natural micro-organisms. Recently the *E. coli* DH1 (pBbA5c-MevTsa-MKco-PMK and pTrc99A-NudB-PMD) strain, capable of isopentenol production, was developed [25]. To demonstrate the automated screening capabilities of our microfluidic platform, we grew the isopentenol-producing *E. coli* in shake-flask cultures, induced production of isopentenol with six different concentrations of IPTG for 48 h, took aliquots of the cultures, and placed the aliquots in the wells of the chip to determine the levels of isopentenol produced using the colorimetric MTBH assay [26].

After the MTBH assay we captured the bright field image of the chip. The isopentenol concentrations were determined based on measurement of pixel intensities within a 21x21 pixel region in the center of each well (blue circles, Additional file 1: Figure S14). Based on the standard curves of the relative intensities, derived from image analysis, we found exponential functions mapping the intensity ratios to the isopentenol concentration and created a curve demonstrating the isopentenol production as a function of IPTG concentration. The chip-based measurements were validated using a conventional plate reader to measure the absorption (at a wavelength of 620 nm) of the MTBH assay samples (Additional file 1: Figure S14).

## Conclusions

Synthetic biology aims to engineer biological systems with desired functions. Construction of these systems is a complex process, often requiring genetic reprogramming, extensive de novo DNA synthesis, and functional screening. The present study was inspired by an approach widely used in engineering disciplines for the development and optimization of new systems, namely the integration of design, construction, testing, and analysis steps (Fig. 1). Adoption of this approach promises to enhance and optimize synthetic biology research, reduce time to product and make the development of biological systems fast, inexpensive, and robust. Herein, we have demonstrated the application of this strategy to synthetic biology research, integrating it with microfluidic technology and laboratory automation.

**Fig. 6:**
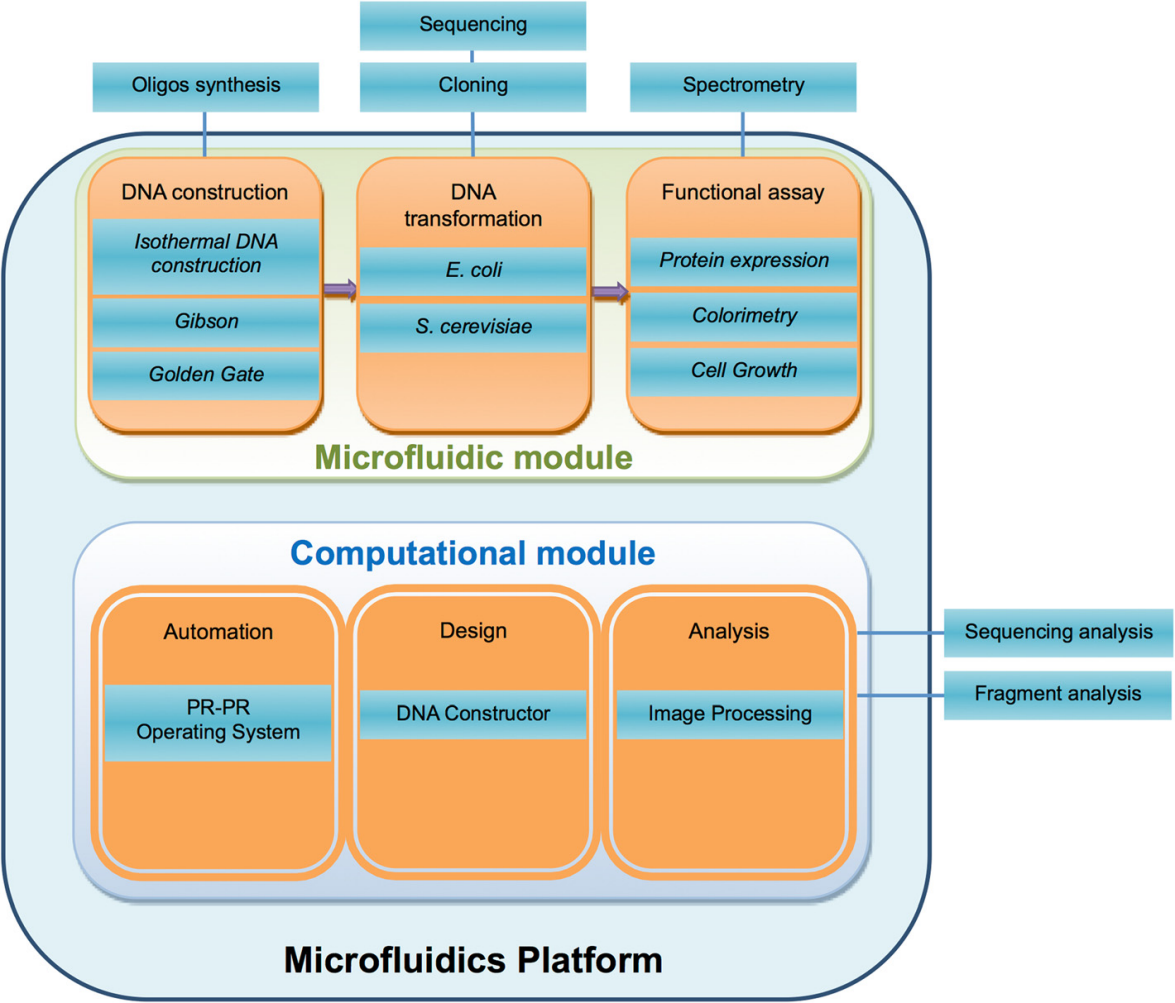
Representation of the microfluidics platform’s functional modules. The platform is built from microfluidic and computational modules

The multipurpose programmable microfluidic platform reported here is a full implementation of the Lab-on-a-Chip paradigm (Fig. 6). We have established the feasibility of the platform by on-chip demonstration of all key steps of the biological product development cycle: design and construction of DNA molecules and combinatorial DNA libraries, subsequent host transformation, induction of protein expression, and screening for desired functionality. The automated protocols implemented on our platform are readily adaptable to a broad range of synthetic biology procedures and host organisms for development of new biological systems, beyond the specific applications to isopentenol production and GFP expression presented here. As part of this work, we report a new software tool for efficient DNA construction design as well as a novel method of de novo DNA assembly, Isothermal Hierarchical DNA Construction (IHDC), specifically developed for the microfluidic environment.

The adaptation of protocols for these operations is facilitated on our platform, as it was especially designed to allow various operations with cells and it has a capability to work in nano- and micro-liter scales. It would potentially cost millions of dollars to purchase traditional liquid handling robots and reagents to perform similar functions at the large (and wasteful) volume scales (see Additional file 1 for time and cost points of comparison with conventional laboratory automation systems). Implementation of the complete process on our multipurpose programmable microfluidic platform, resulting in the desired phenotype, demonstrates its capability to provide an end-to-end solution for synthetic biology research. The ability to perform diverse sample processing operations in a common microfluidic format promotes the integration of microfluidics technology with synthetic biology towards the efficient and robust development of new biological systems.

## Methods

### Microfluidic device fabrication and liquid transfer control

The 32-bit, digitally programmable microfluidic platform was fabricated as a 3-layer glass PDMS (polydimethylsiloxane) hybrid structure. Device features were etched into glass wafers using conventional photolithography and wet chemical etching. Briefly, 1.1 mm-thick 100 mm-diameter borosilicate glass wafers were coated with 200 nm of amorphous polysilicon using low-pressure chemical vapor deposition. The wafers were then spincoated with positive photoresist, soft baked, and patterned with the device design using a contact aligner and a chrome mask. After development and removal of irradiated photoresist, the exposed polysilicon regions were removed by etching in SF6 plasma and the exposed regions of glass were isotropically etched in 49 % hydrofluoric acid to a depth of 70 μm for the pneumatic layer and 30 microns for the fluidic layer. After stripping the remaining photoresist and polysilicon layers, the wafers were diamond-drilled to produce 500 μm-diameter holes for pneumatic input connections. The wafers were then bonded together using a 254 μm-thick PDMS elastomer membrane (HT-6240, Rogers Corporation, Binghamton, NY). To create fluidic reservoirs, holes were punched into 3 mm thick pieces of PDMS, and aligned with each of the sixteen, drilled, fluidic inputs on the array. The use of simpler fabrication methods could open up the technology to more users.

The pneumatically actuated, 2D microvalve array enables discrete transfer of fluids between microvalves within the array. Each monolithic membrane microvalve consists of an etched displacement chamber in the pneumatic layer aligned with a discontinuous microchannel in the fluidic layer. Application of vacuum pulls the PDMS membrane away from the discontinuity, resulting in fluid flow and filling of the microvalves with fluid. Microvalves are actuated by vacuum (-87 kPa) and a closing pressure of 35 kPa is applied to improve the efficiency of the fluidic transfer and mixing operations. Computer controlled solenoid valves were used for delivery of the microvalve actuation pressure.

When transferring reagents to a specific destination, the PR-PR compiler calculates the shortest pathway through the digital 2D microvalve array, represented as graph, and also calculates the number of transfer cycles required for a specific final volume given that each cycle transfers 150 nL. By sequentially opening and closing a series of microvalves in the array, discrete volumes of fluid are transferred through predefined pathways. By iterating this process and increasing the number of cycles, larger volumes are programmably transferred between reservoirs. The rate of transfer is determined by the microvalve actuation time, which can be defined once per reagent or redefined in each transfer command. The fluid transfer rate through the microvalve array depends on reagent properties. For instance, the enzyme mixture requires longer actuation times than water due to its viscosity.

Since multiple distinct source-to-destination fluid transfers may be potentially sequentially routed through the same pathway segment within the device, this opens the possibility of residue from a previous transfer contaminating a subsequent transfer. Particular applications (whether molecular or microbial) could be significantly impacted by small concentrations of contaminants. While in the work presented here, and in previous work [16], cross-contamination does not appear to have impacted our results, this only suggests that contamination was below our threshold of detection. To mitigate cross-contamination concerns for sensitive applications, it would be possible to wash pathways through the device with buffer between reagent transfers. In previous publications, we characterized the valving efficiency and rinsing efficiency for a broad range of sample processing procedures using 2D microvalve array technology, effectively eliminating cross contamination between operations [10, 12, 13].

### DNA constructor

DNA Constructor automatically generates optimized, hierarchical DNA assembly protocols. The application server is written in Python using the Django web framework, and generated protocols are saved in a SQLite3 database hosted on the JBEI server (http://dnaconstructor.jbei.org). DNA Constructor receives as input a nucleotide sequence or set of sequences that the user wishes to assemble, expressed in a novel scripting language that has been developed to support the software. Users can define string variables (DNA Parts) consisting of the DNA alphabet. DNA Constructor allows for the concatenation of string variables with other strings and sub-strings. This allows for the rapid specification of DNA chimeras, mutants, and DNA libraries, in an intuitive and human-readable way (Additional file 1: Figure S6).

DNA is simply expressed as nucleotide strings surrounded by single or double quotation marks. DNA Part declarations come in the form of an unquoted DNA Part name, followed by an equal sign and a DNA sequence or definition. A plus sign between DNA Parts indicates concatenation of DNA sequences. Brackets after a DNA Part name generate a subsequence from the hyphen-separated indices within the brackets. For example, Seq2 = Seq1[5-10] will set the value of the DNA Part Seq2 to nucleotides 5 through 10 (inclusive) of Seq1. To modify (mutate) a subsequence of the sequence in a DNA Part, the construct Part_name([startIndex-endIndex] = DNA) can be used. This will set the sequence of Part_name between the specified start and end indices to DNA sequence, which is either a DNA Part name or string of nucleotides. Finally, the target sequences of an assembly protocol can be set by setting the ‘targets’ variable to a sequence or comma-separated list of sequences.

Upon receiving an input sequence or list of sequences, DNA Constructor uses a novel algorithm to recursively divide the inputs into smaller and smaller pieces until it is left with a list of oligos that are small enough to be synthesized (the threshold for synthesis length is specified by the user). Each target sequence is split into two intermediate assembly fragments, or child sequences. To determine where to divide a sequence into its children, the algorithm begins in the middle of the sequence and works outward. At each potential division point, the software iterates over the possible overlap regions between child sequences (minimum and maximum overlap lengths are specified by the user as well). Each allowed overlap is given a score based on the likelihood of undesirable non-specific sequence interactions as determined by sequence similarity between non-overlap regions of the child fragments. After iterating over these possible overlaps, the algorithm picks the division point and overlap size with the lowest non-specific interaction score. When a division point has been chosen, this same algorithm is applied to the two child sequences created by the division until they are small enough to be synthesized directly. By choosing a division point as close as possible to the center of a DNA sequence fragment, the algorithm creates the most symmetrical assembly tree possible. This results in a protocol involving the lowest total number of reactions, and therefore the least amount of work. When the division algorithm has generated an assembly tree with all the ‘leaf’ sequences small enough to synthesize, it picks primers for every reaction in the protocol. Starting at the root nodes of the tree, DNA Constructor generates primers for the assembly reaction involving the two children of every node. Primers are picked by iterating over the possible primer sizes, as determined by user-specified maxima and minima, and calculating the pairwise alignment score of each potential primer against its template. Primer candidates that have too many matches in their 3’ ends are discarded. As the algorithm moves down the tree, child nodes can inherit either their parent’s forward or reverse primer, depending on which side of the parent sequence they form. This allows for primer re-use in large assembly reactions.

In the case where combinatorial DNA library specified as targets, the software uses a MUSCLE alignment [27] to determine if the targets have any overlapping regions. If a large overlap is found between targets, this overlap sequence is used as an intermediate target in the assembly reaction for all the targets. This minimizes redundancy in the construction of multiple similar target sequences, resulting in reduced synthesis cost and reaction time and effort.

DNA Constructor additionally supports the use of “Natural Fragments” [28], which are meant to represent sequences that the user already has in storage and are available for use as an assembly intermediate. When natural fragments are specified by the user, the division algorithm aligns them with the target sequences to ensure that they are true subsequences of the targets. The software will then attempt to divide the targets in such a way that the Natural Fragments can be used in their entirety as leaf nodes of the assembly tree. This allows users to utilize their existing resources to assemble new pieces of DNA, reducing labor and the potential for errors.

The final result of DNA Constructor is an optimized hierarchical DNA construction protocol and outputs a visual representation of the protocol in the form of an interactive reaction tree (Additional file 1: Figures S3 and S4), implemented using the DOT language used by the Graphviz visualization software [29]. This DOT string is passed on to the client-side Javascript, which uses the canviz.js [30] library to display the tree and allow interactive functionality. Non-leaf nodes in the tree represent individual synthesis reactions, while leaf nodes are oligonucleotides that must be directly synthesized or available natural fragments. Data that are not directly displayed in the tree (such as the primer sequences for each reaction, and the length of the target sequence) are saved in a SQLite database and can be accessed by clicking on individual nodes in the tree or exported and downloaded in FASTA file format.

DNA Constructor is open-source software under the BSD license, is freely available from GitHub https://github.com/JBEI/dna-constructor, and is also available through its web interface on the public DNA Constructor webserver http://dnaconstructor.jbei.org.

DNA Constructor’s database implementation gives it considerable potential to be integrated with other DNA assembly automation platforms, such as j5/DeviceEditor [23, 24] and laboratory automation operation systems such as PR-PR http://prpr.jbei.org [16, 17]. All input scripts used herein for DNA Constructor are available from within Additional file 2. All input scripts used herein for PR-PR are available from within Additional file 3.

### Isothermal hierarchical DNA construction (IHDC)

A basic step of the IHDC on the Microfluidics platform is composed of liquid transfers of the following components to the reactor well (Fig. 4): two DNA fragments from previous steps or oligos (0.2 pmol/μL) 1 μL each, two Primers (0.2 pmol/μL) 2 μL each, mixture of ATP 100 mM (Thermo Scientific) 0.3 μL with magnesium acetate (280 mM) 1.2 μL and enzymes mixture in reaction buffer (TwisDX) 15 μL. Enzyme mixture was prepared by addition of 80 μL rehydration buffer to one tube with lyophilized enzymes. After the reaction assembly the temperature was increased to 38 °C and the reaction is incubated for 15 min. As a result, an elongated and amplified DNA fragment is received and is ready to be used as an input at the next iteration of the IHDC hierarchical process according to the construction tree. The hierarchical construction process is continued this way until the desired DNA molecule is received. The sequences of the DNA primers and oligos used in the construction of *gfp* and *rfp* are shown in Tables 1, 2, 3 and 4.

**Table 1:** *gfp* primers

gfp_Prm_1_F	TTAAGAAGGAGATATAGATATGAGCAAAGGAGAAGAAC
gfp_Prm_2_R	CATAGGTCAGAGTAGTGACAAGTGTTGGCCACGGAACAGGTAGT
gfp_Prm_3_F	CCTGTTCCGTGGCCAACACTTGTCACTACTCTGACCTA
gfp_Prm_4_R	CTTTAACTCGATACGATTAACAAGGGTATCACCTTCAAAC
gfp_Prm_5_F	GTTTGAAGGTGATACCCTTGTTAATCGTATCGAGTTAA
gfp_Prm_6_R	TATTTTGTTGATAATGGTCTGCTAGTTGAACGGAACCA
gfp_Prm_7_F	GATGGTTCCGTTCAACTAGCAGACCATTATCAACAAAATACTCCA
gfp_Prm_8_R	CCCGGGCAGGAATTCGATTTATTTGTAGAGCTCATCCAT

**Table 2:** *gfp* oligos

gfp_Olig_1	TTAAGAAGGAGATATAGATATGAGCAAAGGAGAAGAACTTTTCACTGGAGTTGTCCCAATTCTTGTTGAATTAGATGGTGATGTTAATGGGCACAAATTTTCTGTCCGTGGAGAGGGTGAAGGTGATGC
gfp_Olig_2	CATAGGTCAGAGTAGTGACAAGTGTTGGCCACGGAACAGGTAGTTTTCCAGTAGTGCAAATAAATTTAAGGGTGAGTTTTCCGTTTGTAGCATCACCTTCACCCTCTCCACGGACAGAAAATTTGTGCC
gfp_Olig_3	CCTGTTCCGTGGCCAACACTTGTCACTACTCTGACCTATGGTGTTCAATGCTTTTCCCGTTATCCGGATCACATGAAACGGCATGACTTTTTCAAGAGTGCCATGCCCGAAGGTTATGTACAGGAACGC
gfp_Olig_4	CTTTAACTCGATACGATTAACAAGGGTATCACCTTCAAACTTGACTTCAGCACGCGTCTTGTAGGTCCCGTCATCTTTGAAAGATATAGTGCGTTCCTGTACATAACCTTCGGGCATGGCACTCTTGAAA
gfp_Olig_5	GTTTGAAGGTGATACCCTTGTTAATCGTATCGAGTTAAAGGGTATTGATTTTAAAGAAGATGGAAACATTCTTGGACACAAACTCGAGTACAACTTTAACTCACACAATGTATACATCACGGCAGACAA
gfp_Olig_6	TATTTTGTTGATAATGGTCTGCTAGTTGAACGGAACCATCTTCAACGTTGTGGCGAATTTTGAAGTTAGCTTTGATTCCATTCTTTTGTTTGTCTGCCGTGATGTATACATTGTGTGAGTTAAAGTTGT
gfp_Olig_7	GATGGTTCCGTTCAACTAGCAGACCATTATCAACAAAATACTCCAATTGGCGATGGCCCTGTCCTTTTACCAGACAACCATTACCTGTCGACACAATCTGTCCTTTCGAAAGATCCCAACGAAAAGCGT
gfp_Olig_8	CCCGGGCAGGAATTCGATTTATTTGTAGAGCTCATCCATGCCATGTGTAATCCCAGCAGCAGTTACAAACTCAAGAAGGACCATGTGGTCACGCTTTTCGTTGGGATCTTTCGAAAGGACAGATTGTGTC

**Table 3:** *rfp* primers

rfp_Prm_1_F_int	ttaagaaggagatatagatATGGCGAGTAGCGAAGACGTTATCAAAGAGTTCATGCG
rfp_Prm_2_R	TAGATGAACTCACCGTCTTGCAGGGAGGAGTCCTGGGTAACG
rfp_Prm_3_F	TTACCCAGGACTCCTCCCTGCAAGACGGTGAGTTCATC
rfp_Prm_4_R_int	cccgggcaggaattcgatTTAAGCACCGGTGGAGTGACGACCTTCAGCACGTTCGT

**Table 4:** *rfp* oligos

rfp_Olig_1	ATGGCGAGTAGCGAAGACGTTATCAAAGAGTTCATGCGTTTCAAAGTTCGTATGGAAGGTTCCGTTAACGGTCACGAGTTCGAAATCGAAGGTGAAGGTGAAGGTCGTCCGTACGAAGGTACCCAGACCGCTAAACTGAAAGTTACCAAAGGTGGTCCGCTGCCGTTCGCTTGGGACATCCTGTCCCCGCAGTTCCAGT
rfp_Olig_2	TAGATGAACTCACCGTCTTGCAGGGAGGAGTCCTGGGTAACGGTAACAACACCACCGTCTTCGAAGTTCATAACACGTTCCCATTTGAAACCTTCCGGGAAGGACAGTTTCAGGTAGTCCGGGATGTCAGCCGGGTGTTTAACGTAAGCTTTGGAACCGTACTGGAACTGCGGGGACAGGATGTCCCAAGCGAACGGCAG
rfp_Olig_3	TTACCCAGGACTCCTCCCTGCAAGACGGTGAGTTCATCTACAAAGTTAAACTGCGTGGTACCAACTTCCCGTCCGACGGTCCGGTTATGCAGAAAAAAACCATGGGTTGGGAAGCTTCCACCGAACGTATGTACCCGGAAGACGGTGCTCTGAAAGGTGAAATCAAAATGCGTCTGAAACTGAAAGACGGTGGTCACTA
rfp_Olig_4	TTAAGCACCGGTGGAGTGACGACCTTCAGCACGTTCGTACTGTTCAACGATGGTGTAGTCTTCGTTGTGGGAGGTGATGTCCAGTTTGATGTCGGTTTTGTAAGCACCCGGCAGCTGAACCGGTTTTTTAGCCATGTAGGTGGTTTTAACTTCAGCGTCGTAGTGACCACCGTCTTTCAGTTTCAGACGCATTTTGATTT

### DNA plasmids

All DNA plasmids used in this study (Table 5) are available from the JBEI public registry [31] in collection https://public-registry.jbei.org/folders/160.

**Table 5:** Plasmids used in this study

JBEI Registry ID	Name	Details	Source
JPUB_004942	pETBlue-RFP	RFP cloned in pETBlue vector in the EcoRV cloning site	This study
JPUB_004941	pETBlue-GFP	GFP cloned in pETBlue vector in the EcoRV cloning site	This study
JPUB_004940	pRS426-yeGFP	pRS426 shuttle vector with URA3 marker and *gfp*	JBEI
JPUB_004949	pRS426-Ptef1-100-yeGFP	pRS426 with GFP and Ptef1-100 promoter	This study
JPUB_004948	pRS426-Ptef1-250-yeGFP	pRS426 with GFP and Ptef1-250 promoter	This study
JPUB_004947	pRS426-Pspo13-100-yeGFP	pRS426 with GFP and Pspo13-100 promoter	This study
JPUB_004946	pRS426-Pspo13-250-yeGFP	pRS426 with GFP and Pspo13-250 promoter	This study
JPUB_004945	pRS426-Pleu2-250-yeGFP	pRS426 with GFP and Pleu2-250 promoter	This study
JPUB_004944	pRS426-Pgal1-100-yeGFP	pRS426 with GFP and Pgal1-100 promoter	This study
JPUB_004943	pRS426-Pgal1-250-yeGFP	pRS426 with GFP and Pgal1-250 promoter	This study
JPUB_004939	pETBlue-1	The pETBlue vector allows blue/white screening and also has T7lac promoter for expression of target genes	Novagen
JPUB_004964	pProm11_BCD1-GFP	Promoter11 with BCD1-gfp	[16]
JPUB_004963	pProm9_BCD1-GFP	Promoter9 with BCD1-gfp	[16]
JPUB_004962	pProm2_BCD1-GFP	Promoter2 with BCD1-gfp	[16]
JPUB_004961	pProm1_BCD21-GFP	Promoter1 with BCD21-gfp	[16]
JPUB_004960	pProm1_BCD20-GFP	Promoter1 with BCD20-gfp	[16]
JPUB_004959	pProm1_BCD2-GFP	Promoter1 with BCD2-gfp	[16]
JPUB_004958	pProm1_BCD1-GFP	Promoter1 with BCD1-gfp	[16]
JPUB_004957	pProm11_BCD21-GFP	Promoter11 with BCD21-gfp	[16]
JPUB_004956	pProm11_BCD20-GFP	Promoter11 with BCD20-gfp	[16]
JPUB_004955	pProm11_BCD2-GFP	Promoter11 with BCD2-gfp	[16]
JPUB_004954	pProm9_BCD21-GFP	Promoter9 with BCD21-gfp	[16]
JPUB_004953	pProm9_BCD20-GFP	Promoter9 with BCD20-gfp	[16]
JPUB_004952	pProm9_BCD2-GFP	Promoter9 with BCD2-gfp	[16]
JPUB_004951	pProm2_BCD21-GFP	Promoter2 with BCD21-gfp	[16]
JPUB_004950	pProm2_BCD2-GFP	Promoter2 with BCD2-gfp	[16]
JPUB_004950	pProm2_BCD20-GFP	Promoter2 with BCD20-gfp	[16]
JPUB_004938	pTrc99A-NudB-PMD	PMD cloned downstream NudB	[25]
JPUB_004937	MevTsa-MKco-PMKco	MevTsa with MKco-PMKco	[25]

### Digestion of pETBlue-1 plasmid by EcoRV

Four 20 μL EcoRV digestion reactions each consisting of 15 μL purified plasmid pETBlue-1 (100 ng/μL), 2 μL CutSmart™ Buffer, 1 μL EcoRV-HF (NEB) (20 units/μL), and 2 μL deionized water were assembled on chip. The plasmid was loaded into four input wells and the reaction mixture of enzyme buffer and water were transferred automatically on-chip and mixed with the plasmid. The digestion mixture was incubated for 1 h at 37 °C. Digested samples were combined and purified by PCR purification kit (Qiagen) according to the manufacturer’s protocol.

### Gibson assembly

We have adapted the Gibson DNA assembly method to our microfluidic platform and integrated it with our IHDC method. The output DNA fragments of IHDC were designed to be compatible with the Gibson method. We showed construction of pETBlue-GFP and pETBlue-RFP plasmids on our platform. For two-fragment assembly, we loaded either two halves of gfp or full-length rfp constructed by IHDC, and digested pETBlue-1 plasmid, into the device’s input wells. We transferred automatically to the reaction well 2 μL of each fragment, 1 μL of digested backbone, and 5 μL of Gibson Assembly mix (New England Biolabs). The scheme of the automated program for Gibson assembly is shown in Additional file 1: Figure S8. After the assembly reactions were prepared, the reactions were incubated at 50 °C for 30 min on-chip. Circularized pETBlue-GFP and pETBlue-RFP plasmids containing de novo synthesized GFP and RFP DNA fragments, ready for transformation, result.

### Transformation to E. coli

We have transformed the newly assembled pETBlue-GFP and pETBlue-RFP plasmids into *E. coli* host cells Tuner (DE3) pLacI (Novagen). For each transformation 14 μL of the chemically competent *E. coli* cells and 1 μL of the Gibson assembly mixture (i.e., pETBlue-GFP or pETBlue-RFP plasmids) were loaded into the microfluidic chip when it was cooled down to 0 °C. The competent *E. coli* cells were transferred to the wells containing the DNA plasmids (Additional file 1: Figure S9). The DNA and the cells were incubated for 10 min at 0 °C and then the heat shock was performed at 42 °C for 45 s. Then the transformation mixture was cooled to room temperature and the cells were incubated off-chip with 100 μL SOC medium for a half an hour at 37 °C. Ultimately all the cells were plated on LB-Amp agar plates and incubated at 37 °C overnight to produce colonies of transformed *E. coli* containing desired plasmids.

### Golden gate assembly

We have previously completed the construction of a 16-variant DNA library on microfluidics platforms [16]. We amplified all the DNA fragments, defined in the library: Promoters, BCD_GFP and Plasmid backbone by PCR. The methylated templates (plasmids) were digested by DpnI. All the amplified fragments designed with recognition sites for BsaI. We digested the ends of the fragments by BsaI and used these digested fragments with sticky ends to construct a full combinatorial library using the combinatorial assembly protocol (Additional file 1: Figure S10) on our programmable microfluidic platform. We created a protocol in PR-PR that describes reagents flow for construction of combinatorial library with two variable fragments and one shared fragment by the Golden Gate assembly method. Each reaction contains three components: 1 μL BsaI-digested promoter fragment, 1 μL BsaI-digested BCD variant fragment and 8 μL ligation reaction master mix containing 1 μL BsaI-digested vector backbone, 1 μL of T4 ligase enzyme (Thermo Scientific), 1 μL of T4 ligase buffer, and 5 μL deionized water. The reactions were incubated for 30 min at room temperature. The chip was preloaded with ligation reaction master mix in each reaction wells and the promoters and BCD were transferred automatically according the protocol in combinatorial way. Given the limited number of input and output wells available on the microfluidic device, we executed the microfluidic DNA assembly protocol twice, first assembling the first 8 constructs (pProm1_BCD1-GFP … pProm2_BCD21-GFP) and then assembling the last 8 constructs (pProm9_BCD1-GFP … pProm11_BCD21-GFP).

### MBTH assay

To demonstrate an automated microbial product screening assay using our microfluidics platform, we grew E. coli DH1 harboring plasmids pBbA5c-MevTsa-MKco-PMK and pTrc99A-NudB-PMD [25], induced the production of isopentenol by adding different levels of IPTG, and measured on-chip the isopentenol concentration using a colorimetric MBTH assay. For the MBTH assay, 3 mg/ml 3-methyl-2-benzothiazol-inone hydrazone hydrochloride hydrate (MBTH) solution, and acid solution (5 mg/mL sulfamic acid and 5 mg/mL ammonium iron (III) solfate dodecahydrate), were prepared. The isopentenol-production cell culture samples were inoculated when OD600 reached 0.4 and induced with IPTG to a final concentration ranging from 0.01 μM to 1 μM. 48 h after induction, cell cultures were centrifuged to obtain the supernatant. 10 μL of the supernatants of each of the six culture samples, and MBTH solution and acid solution were loaded to designated wells of the microfluidic platform, allowing automated programed serial reactions. Firstly, 4 μL MBTH solution was transferred to the sample wells, incubated at room temperature for 15 min. 10 μL acid solution was then added to this mixture. After 20 min we observed development of blue color of various intensities, indicating production of isopentenol. After the two incubations, we took a picture of the chip for image analysis (Additional file 1: Figure S14) and also we took 2 μL samples of the mixture and measure absorbance at 620 nm using the Nanodrop (Thermo Fisher Scientific). The same protocol was also used to obtain readings for the isopentenol standards. The isopentenol standards were prepared by 5 times two-fold serial dilutions from 125 mg/L to 3.9 mg/L.

### Amplification of yeast promoters

Template preparation: we picked a small colony of *S. cerevisiae* by sterile pipette tip and washed it in 10 uL of 0.02 M NaOH in a PCR tube. The sample was boiled for 10 min at 99 °C. Amplification conditions: 50 μL PCR reactions consisted of 2.5 μL (2.5 μM) of each forward and reverse primer, 1 μL template, 1 μL dNTPs (10 mM), 0.5 μL high fidelity phusion polymerase (Thermo Fisher Scientific), 10 μL 5× high fidelity phusion buffer, and 32.5 μL deionized water. PCR thermocycling conditions were used: denaturation at 98 °C for 30 s, 30 cycles of denaturation at 98 °C for 20s, annealing at 60 °C for 20 s, and elongation at 72 °C for 30 s and a final extension at 72 °C for 3 min. The primers are shown in Table 6.

**Table 6:** DNA primers used to amplify yeast promoters

Pgal1-250 F	GGATCCACTAGTTCTAGAGCGGCCGCCACCGCGGTGGAGCACGAATCAAATTAACAACCA
Pgal1-100 F	GGATCCACTAGTTCTAGAGCGGCCGCCACCGCGGTGGAGCATTTTCAGTTTGTATTACTT
Pgal1-R	TTGGGACAACACCAGTGAATAATTCTTCACCTTTAGACATTATAGTTTTTTCTCCTTGAC
Pleu2-250 F	GGATCCACTAGTTCTAGAGCGGCCGCCACCGCGGTGGAGCGCATATACCTTTTTCAACTG
Pleu2-100 F	GGATCCACTAGTTCTAGAGCGGCCGCCACCGCGGTGGAGCTTTTCCAATAGGTGGTTAGC
Pleu2-R	TTGGGACAACACCAGTGAATAATTCTTCACCTTTAGACATTAGAATGGTATATCCTTGAA
Pspo13-250 F	GGATCCACTAGTTCTAGAGCGGCCGCCACCGCGGTGGAGCTATTTACACATCTAATTTTT
Pspo13-100 F	GGATCCACTAGTTCTAGAGCGGCCGCCACCGCGGTGGAGCAAATAGCCGCCGACAAAAAG
Pspo13-R	TTGGGACAACACCAGTGAATAATTCTTCACCTTTAGACATAATTATTCTCGACTCAACTT
Ptef1-250 F	GGATCCACTAGTTCTAGAGCGGCCGCCACCGCGGTGGAGCAAAAGAGACCGCCTCGTTTC
Ptef1-100 F	GGATCCACTAGTTCTAGAGCGGCCGCCACCGCGGTGGAGCTCAAGTTTCAGTTTCATTTT
Ptef1-R	TTGGGACAACACCAGTGAATAATTCTTCACCTTTAGACATTTTGTAATTAAAACTTAGAT

### Digestion of pRS426-yeGFP plasmid by EagI

50 μL EagI (NEB) digestion reactions consisting of 20 μL purified plasmid pRS426 (100 ng/μL), 5 μL CutSmart™ Buffer, EagI-HF (20 units/μL) 1 μL and 24 μL deionized water. The digestion mixture was incubated for 1 h at 37 °C. EagI was deactivated at 65 °C for 20 min. Digested samples were purified by PCR purification kit (Qiagen) according to manufacturer’s protocol.

### Assembly and transformation to yeast

As a backbone for in vivo DNA construction, we used plasmid pRS426, which was linearized on-chip by EagI restriction enzyme. After restriction the purified plasmid and each purified promoter amplicons separately were mixed with Salmon sperm DNA as a carrier (Clontech). The yeast cells were grown overnight in 100 ml 2xYPAD medium on a rotary shaker at 200 rpm and 30 °C, washed twice in sterile water and re-suspended in 25 % PEG 400 and 0.1 M lithium acetate (LiAc) solution. The DNA mixture and cell solution were loaded to the input wells on-chip. The competent *S. cerevisiae* cells were transferred to wells with preloaded DNA mixture atomically according to the PR-PR protocol. After the cell transfer, the chip was incubated for two hours at 42 °C. After the incubation, the *S. cerevisiae* cells were plated onto rich medium lacking uracil and cultured overnight to produce colonies of cells expressing GFP.

### Image analysis

#### Image analysis for phenotype screening assay

After construction of gfp and transformation into *E. coli*, we estimated the GFP expression levels by the engineered *E. coli* cells and analyzed the image of the chip with 6 wells containing induced cells and 6 wells containing non-induced cells. We measured the intensity average over squares of size 21x21 pixels with centers in wells (green circles) and then calculated the relative intensities in linear space of the induced and non-induced wells. We used only the Green value of (R,G,B). The average brightness for induced wells was 0.326 and for non-induced wells was 0.0377, an 8.6-fold difference. The observed brightness data is shown in Table 7. Note that while this image analysis (and that described immediately below for the MTBH assay) both utilize pixel intensities (scalar values), it would be possible to implement a colorimetric assay by comparative analysis of the intensities of each R/G/B channel (vector values).

**Table 7:** Observed brightness data for on-chip phenotype screening assay

Induced	
Well Num.	Brightness
1	0.3527
3	0.3623
5	0.3003
7	0.3047
9	0.3138
11	0.3220
Average	0.3260
Non-induced	
Well Num.	Brightness
2	0.0298
4	0.0433
6	0.0273
8	0.0421
10	0.0394
12	0.0445
Average	0.0377

### Image analysis for MTBH assay

After the MTBH assay, we captured bright field image of the chip. To determine isopentenol concentrations, produced by *E. coli* after the IPTG induction, based on analysis of pixel intensities, we analyzed the image of the chip after MTBH assay and measured the intensities within a 21x21 pixel region in the center of each well (blue circles, Additional file 1: Figure S14). Based on image analysis we have built the MTBH assay calibration curve: we found exponential function mapping the pixel intensity ratios to the isopentenol concentrations (IC). The function is IC = 6*10^-19^*e^45.972*(1-intesity)^. The calibration curve data is shown in Table 8. The results of the MTBH assay experiment are shown in Table 9.

**Table 8:** Calibration curve data for on-chip isopentenol MTBH assay

Intensity	Isopentenol (mg/L)
0.0682	3.91
0.0287	7.81
0.0162	15.6
0.0086	31
0.0043	63
0.0035	130

**Table 9:** Experiment results for on-chip isopentenol MTBH assay

IPTG μM	Intensity	Isopentenol mg/L
0.01	0.2028	0.0178
0.05	0.1011	1.911
0.1	0.0575	14.2
0.2	0.0437	26.8
0.5	0.0155	97.8
1	0.0077	140

Based on this mapping function we then calculated the isopentenol concentration received from induced production of isopentenol in *E. coli* strain by different levels of IPTG: we measured the pixel intensities from the image received after the MTBH assay on-chip and calculated the isopentenol concentrations using the exponential function, factoring by 3.6 to compensate the image brightness. Then we compared the results of our image analysis method to a well-defined method of isopentenol concentrations measurement, using OD620 absorbance after the MTBH assay: based on a calibration curve we have found a function mapping the OD620 to isopentenol concentrations - IC = 3.2809e^7.4512*OD620^ (Table 10) and calculated the actual isopentenol concentrations received after IPTG induction (Table 11). Both measurement methods have shown similar correlation between IPTG and isopentenol production by *E. coli*.

**Table 10:** Calibration curve data for off-chip isopentenol MTBH assay

OD620	Isopentenol (mg/L)
0.052	3.9
0.101	7.81
0.188	15.6
0.296	31.3
0.412	62.5
0.487	125

**Table 11:** Experiment results for off-chip isopentenol MTBH assay

IPTG μM	OD620	Isopentenol mg/L
0.01	0.014	3.6
0.05	0.079	5.9
0.1	0.127	8.45
0.2	0.179	12.5
0.5	0.456	98.1
1	0.506	142

### Experiment setup and workflow

1. Analysis of experiment and implementation of its protocol as PR-PR script
  - Division of protocols into procedures and into basic transfer steps
  - Writing the PR-PR script and compilation into microfluidics program
2. Preparation of the microfluidics chip
  - Washing the chip with water
  - Loading the chip with reagents according to the PR-PR script
3. Running the experiment
  - Automated execution of the experiment: reagents transfer, incubation, and image capture
  - Collection of processed samples from the chip

## Additional files

Additional file 1:
**Supplementary information.pdf. Figure S1.** Platform schematic. **Figure S2.** Valve actuation and peristaltic liquid movement. **Figure S3.** Construction of *gfp.* **Figure S4.** Construction of *rfp.* **Figure S5.** Representative DNA construction tree. **Figure S6.** Representative DNA Constructor syntax. **Figure S7.** EcoRV digestion of pETBlue < 1 plasmid. **Figure S8.** Gibson Assembly. **Figure S9.** Transformation. **Figure S10.** Golden Gate combinatorial DNA library construction. **Figure S11.** Yeast promoter library transformation and assessment. **Figure S12.** Screen for GFP < positive clones following pETBLUE < GFP transformation into *E. coli.* **Figure S13.** Induction of GFP expression on < chip. **Figure S14.** MBTH assay. **Figure S15.** Demonstration of a complete process imparting a desired function into *E. coli.* (PDF 3879 kb) Additional file 2:
**DNAConstructor scripts.zip.** Zip file containing files gfp_DNAConstructor.txt and rfp_DNAConstructor.txt, input script files for DNA Constructor. (ZIP 1.7 kb) Additional file 3:
**PR-PR scripts.zip.** Zip file containing files Gibson_Assembly.pr, IHDC_1.pr, IHDC_2.pr, IHDC_3.pr, Induction.pr, MBTH.pr and Transformation.pr, input script files for PR-PR. (ZIP 5.73 kb)
