# Supplementary material for: End-to-end automated microfluidic platform for synthetic biology: from design to functional analysis
Source: J Biol Eng. 2016 Feb 2;10:3. doi: 10.1186/s13036-016-0024-5 (PMC4736182; doi:10.1186/s13036-016-0024-5)
Supplement: Additional file 1: — Supplementary information.pdf. Figure S1. Platform schematic. Figure S2. Valve actuation and peristaltic liquid movement. Figure S3. Construction of gfp. Figure S4. Construction of rfp. Figure S5. Representative DNA construction tree. Figure S6. Representative DNA Constructor syntax. Figure S7. EcoRV digestion of pETBlue < 1 plasmid. Figure S8. Gibson Assembly. Figure S9. Transformation. Figure S10. Golden Gate combinatorial DNA library construction. Figure S11. Yeast promoter library transformation and assessment. Figure S12. Screen for GFP < positive clones following pETBLUE < GFP transformation into E. coli. Figure S13. Induction of GFP expression on < chip. Figure S14. MBTH assay. Figure S15. Demonstration of a complete process imparting a desired function into E. coli. (PDF 3879 kb) [file 13036_2016_24_MOESM1_ESM.pdf]

Supplementary information and figures

Time and cost points of comparison with conventional laboratory automation systems

Time comparisons

Although the end-to-end microfluidic platform described here can reduce hands-on time at the bench (just as conventional laboratory automation systems do), overall process time is typically limited by reaction/incubation times, and microorganism growth rates. For many applications, overall process time is therefore expected to be similar across conventional laboratory automation systems, manual pipetting techniques, and our microfluidic automation platform. That said, shorter process step times may be enabled by the microfluidic platform, for example sample temperature can be changed more rapidly at smaller sample volumes. See below for a discussion of minimum sample volume requirements for the microfluidic and conventional automation platforms.

Cost comparisons

Disposables. The microfluidic platform eliminates the need for disposable pipette tips, except for the initial loading of the device and for extracting processed samples (optional) from the device. This contrasts with both manual methods and conventional laboratory automation systems that require an additional pipette tip for each fluidic transfer step. The microfluidic platform also reduces the need for other disposables such as microfuge tubes and micro-titer plates.

Platform. The control equipment for a 32-bit microfluidic processor (similar to that used for the microfluidic platform described here) can be assembled for less than \$2000. In contrast, conventional laboratory robots can cost hundreds of thousands of dollars.

Device. Although the microfluidic device described here was fabricated in glass, a costly and laborious procedure, similar microvalve-based devices could be fabricated using thermoplastic materials including Teflon (1), PMMA (2), and COC (3) for the pneumatic and fluidic layers instead of glass. The use of these materials enables higher throughput manufacturing methods such as embossing or injection molding, which could reduce the cost of the microfluidic chips to less than a few dollars per device (or even less depending on manufacturing scale). Furthermore, efficient rinsing programs (4-6) enable device reuse for many applications.

Sample volume requirements. In the work described here, we used sample and reagent volumes ranging from 150 nL to 20 uL. The key advantage of our microfluidic platform is the broad range of sample volumes that can be precisely metered. In previous work, we have demonstrated precise control over sample metering operations involving as little as 40 nL (5). Precisely controlled biochemical reactions have been demonstrated by metering and mixing samples and reagents within a 120 nL microvalve (4). This demonstrates precise sample processing at a volume scale approximately one order of magnitude lower than conventional sample handling robots (typically > 2 uL). Our platform can therefore reduce sample volume costs by up to an order of magnitude compared to conventional robots and manual sample processing.

Labor. The reduction in labor costs associated with our platform is expected to be equivalent to that of conventional laboratory automation robots.

References

1. Ren K, Dai W, Zhou J, Su J, Wu H. Whole-Teflon microfluidic chips. Proc Natl Acad Sci U S A. 2011;108(20):8162-6.
2. Zhang W, Lin S, Wang C, Hu J, Li C, Zhuang Z, et al. PMMA/PDMS valves and pumps for disposable microfluidics. Lab on a chip. 2009;9(21):3088-94.
3. Novak R, Ranu N, Mathies RA. Rapid fabrication of nickel molds for prototyping embossed plastic microfluidic devices. Lab on a chip. 2013;13(8):1468-71.
4. Jensen EC, Bhat BP, Mathies RA. A digital microfluidic platform for the automation of quantitative biomolecular assays. Lab on a chip. 2010;10(6):685-91.
5. Jensen EC, Stockton AM, Chiesl TN, Kim J, Bera A, Mathies RA. Digitally programmable microfluidic automaton for multiscale combinatorial mixing and sample processing. Lab on a chip. 2013;13(2):288-96.
6. Jensen EC, Zeng Y, Kim J, Mathies RA. Microvalve Enabled Digital Microfluidic Systems for High Performance Biochemical and Genetic Analysis. Jala. 2010;15(6):455-63.

Figure S1. Platform schematic

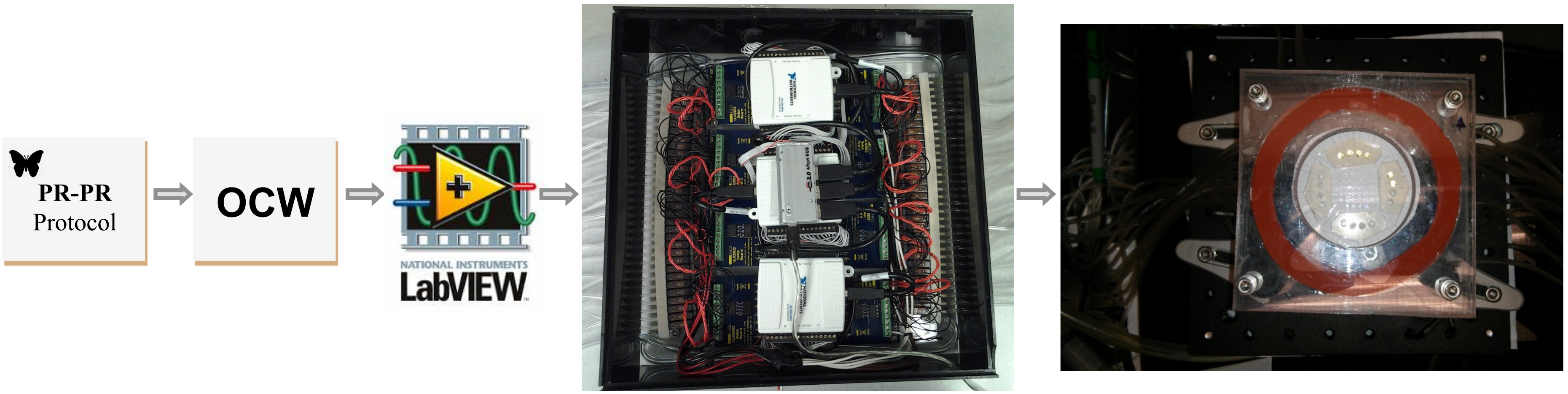

Supplementary Figure 1. Platform schematic. The platform is managed by PR-PR, a web-based high-level programing language for laboratory automation, that translates user-defined sample processing operations into a sequence of operational commands for microvalve Open Close Wait (OCW) control. The PR-PR output script is processed by LabView, which transmits the operational commands to an array of miniature solenoid valves. Each solenoid valve switches between positive pressure (closing) and vacuum (opening) outputs, and controls a unique microvalve within the 2D array.

Figure S2. Valve actuation and peristaltic liquid movement

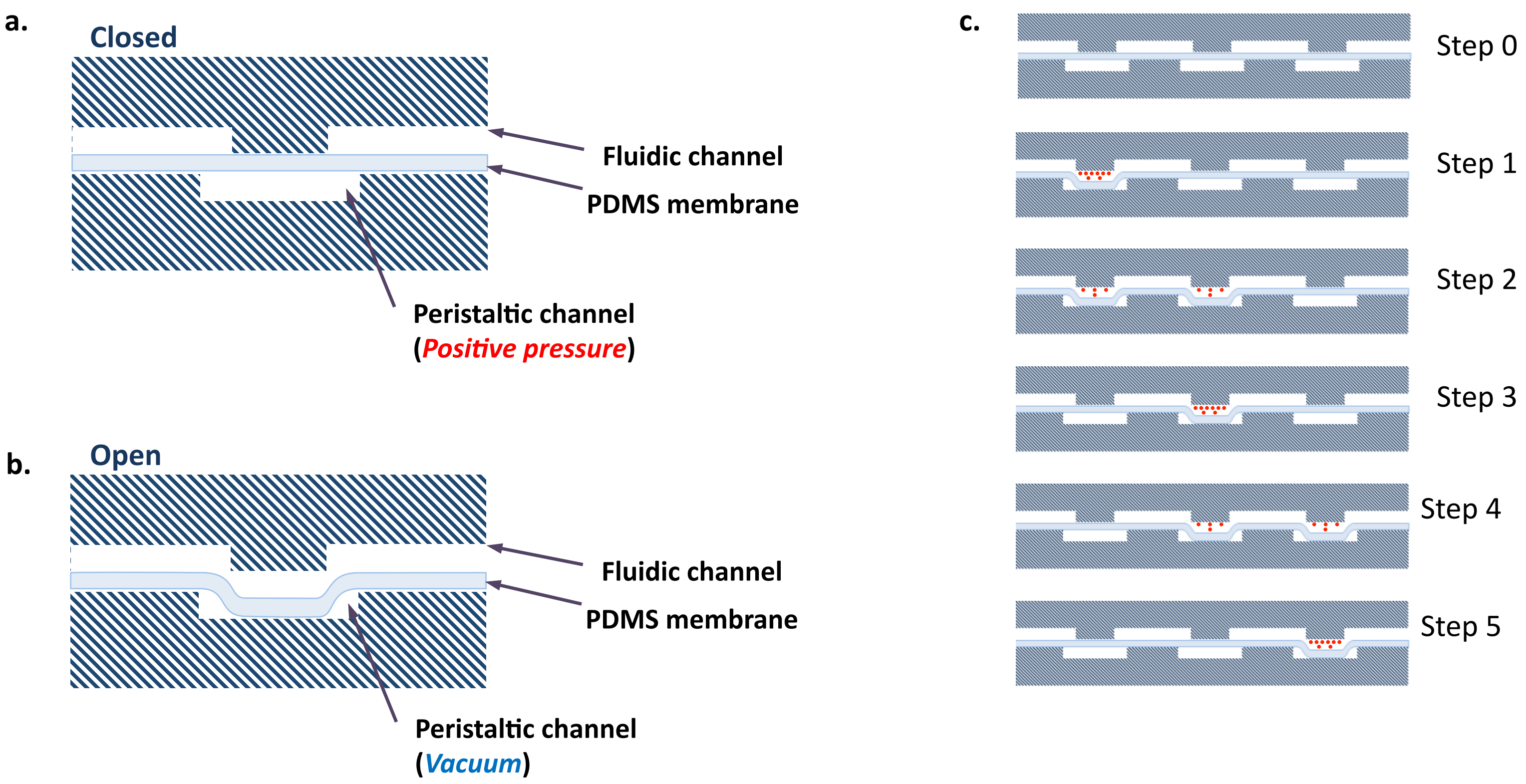

Supplementary Figure 2. Valve actuation and peristaltic liquid movement. (a) Applied positive pressure closes valve. **(b)** Applied vacuum opens valve. **(c)** Scheme of peristaltic liquid movement by sequential opening and closing the valves.

Figure S3. Construction of *gfp*

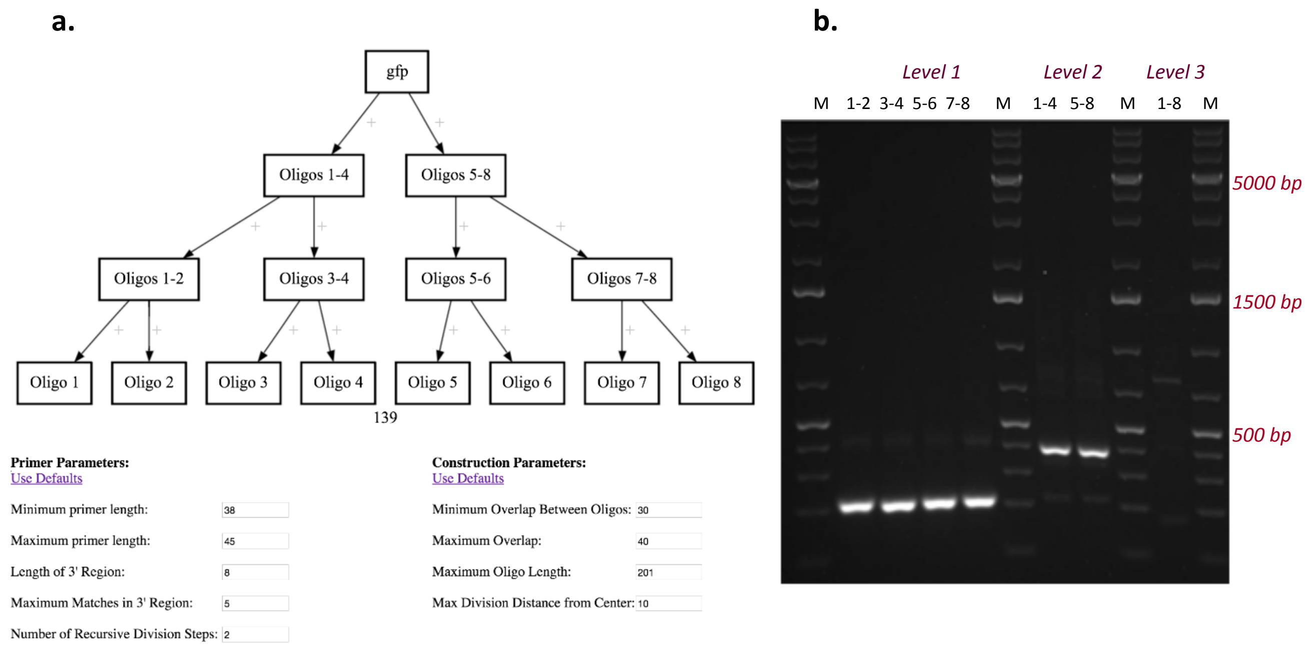

Supplementary Figure 3: Construction of *gfp*. (a) DNA Constructor protocol design. **(b)** Gel electrophoresis image of IHDC construction of *gfp.* Lanes as labelled by: M: GeneRuler 1 kb Plus DNA Ladder (Thermo Scientific); Level 1 (quarter) fragments: 1-2, 3-4, 5-6, 7-8; Level 2 (half) fragments: 1-4 and 5-8; Level 3 (full length *gfp*) fragment: 1-8.

Figure S4. Construction of *rfp*

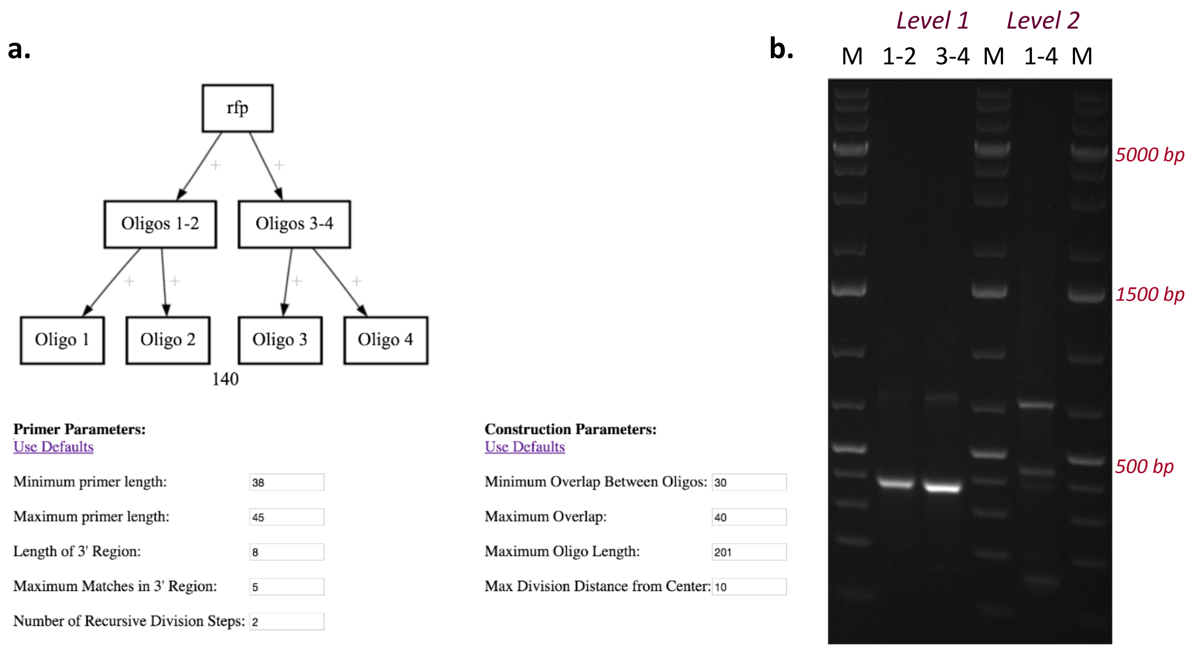

Supplementary Figure 4: Construction of *rfp*. (a) DNA Constructor protocol design. (b) Gel electrophoresis image of IHDC construction of *rfp*. Lanes as labelled by: M: GeneRuler 1 kb Plus DNA Ladder (Thermo Scientific); Level 1 (half) fragments: 1-2 and 3-4; Level 2 (full length *rfp*) fragment: 1-4.

Figure S5. Representative DNA construction tree

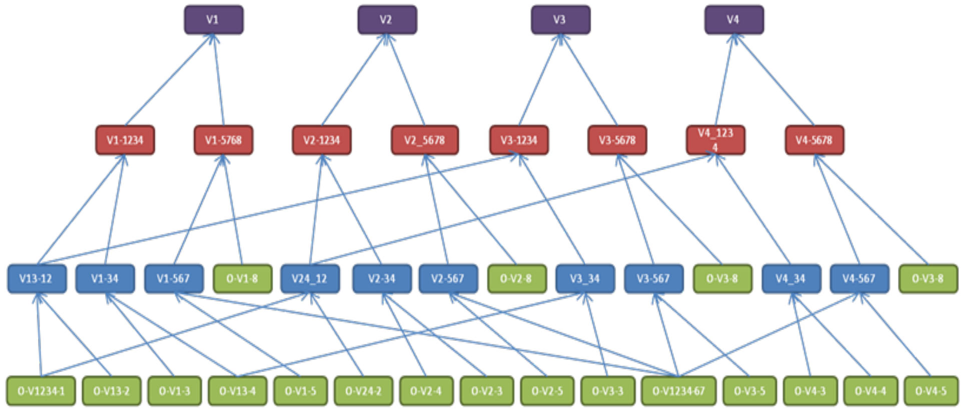

Supplementary Figure 5: Representative optimized DNA Constructor hierarchical DNA construction tree that re-uses shared components between variants.

Figure S6. Representative DNA Constructor syntax

```
Seq1='ATGAGCAAAGGAGAAGAACTTTTCACTGGAGTTGTCCCAAT'
Seq2='GAAGGTGATGCTACAAACGGAAAACTCACCCTTAAATTTATTTGC'

Targ1 = Seq1[0-10] + 'ACG' + Seq2[5-15]
Targ2 = Seq1[0-10] + Seq2[10-17] + 'CGT'
Targ3 = Targ1 + Targ2
Targ4 = Targ1 + Targ3
Targ5 = Seq1([8-15]="AAACAAA")

targets = Targ1, Targ2, Targ3, Targ4, Targ5
```

Supplementary Figure 6: Representative DNA Constructor syntax. Targ1 is the concatenation of subsequences of Seq1 and Seq2 with ACG inserted in between the subsequences; Targ2 is CGT appended to the concatenation of subsequences of Seq1 and Seq2; Targ3 and Targ4 are the concatenations of intermediate sequences Targ1-3; Targ5 is a variant of Seq1 with sequence positions 8 to 15 replaced by AAACAAA; targets defines the final sequence candidates to be synthetized.

Figure S7. EcoRV digestion of pETBlue-1 plasmid

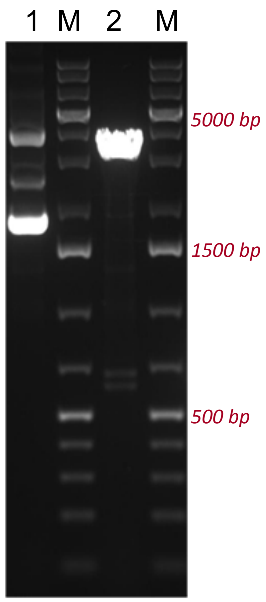

Supplementary Figure 7: Gel electrophoresis image of EcoRV digestion of pETBlue-1 plasmid. Lane 1: pETBlue-1 before digestion; Lane 2: pETBlue-1 plasmid digested by EcoRV; M is GeneRuler 1 kb Plus DNA Ladder (Thermo Scientific).

Figure S8. Gibson Assembly

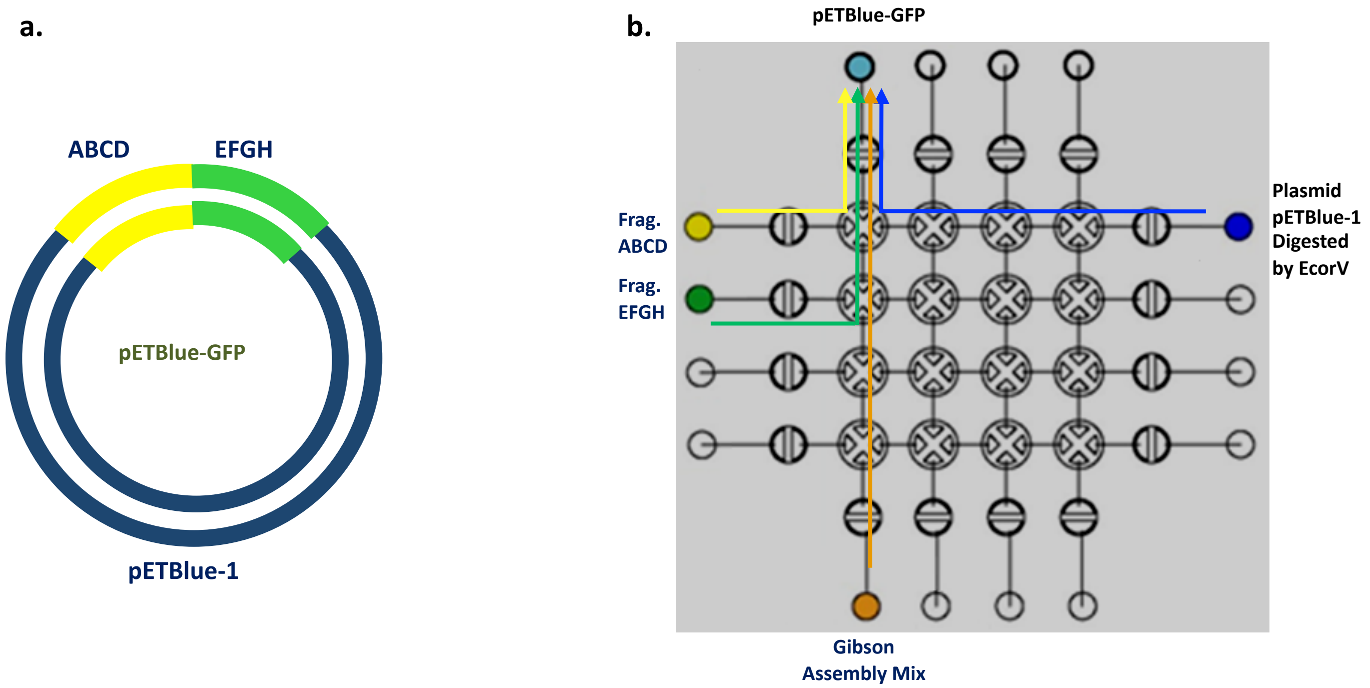

Supplementary Figure 8: Gibson Assembly. (a) Design assembling two split halves of *gfp* together with pETBlue-1 plasmid backbone digested by EcoRV. **(b)** Schematic of reagent transfers through the microfluidic chip.

Figure S9. Transformation

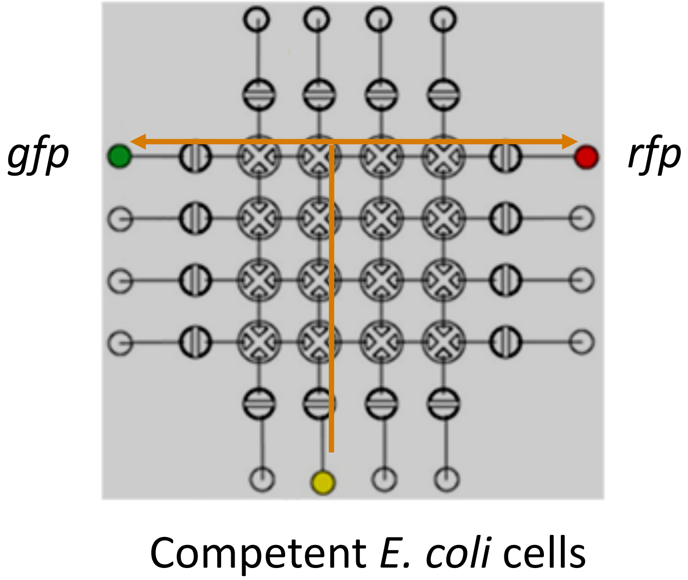

Supplementary Figure 9: Schematic of transformation of *gfp* and *rfp* DNA into competent *E. coli* cells.

Figure S10. Golden Gate combinatorial DNA library construction

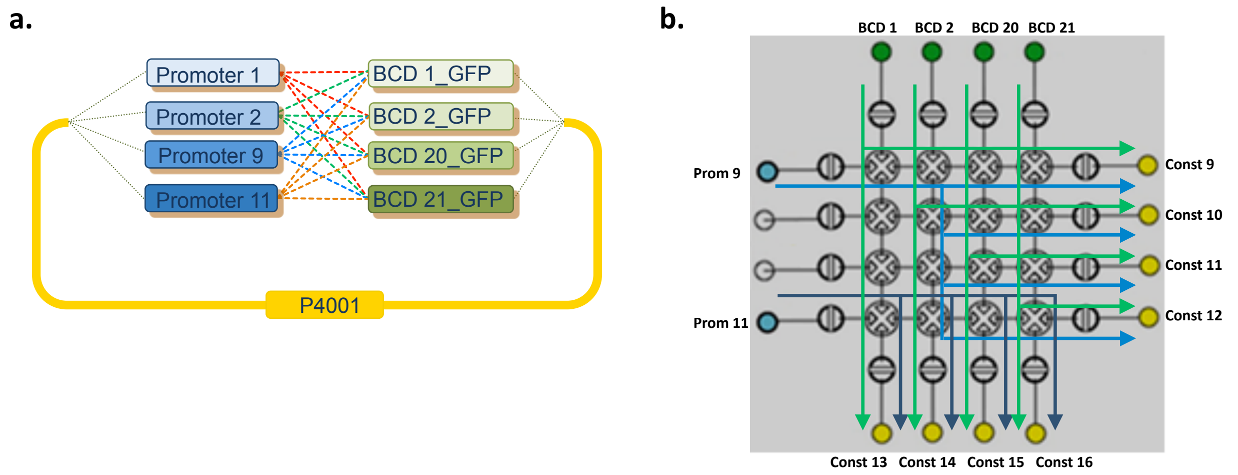

Supplementary Figure 10: Golden Gate combinatorial DNA library construction. (a) Schematic of the 16 construct library. **(b)** Schematic of the microfluidics platform configuration for the automated construction of 8 of the 16 variants. The arrows show patches of the reagents transfer on-chip according to the automated protocol (Linshiz, G., Stawski, N., Goyal, G., Bi, C., Poust, S., Sharma, M., Mutalik, V., Keasling, J.D., and Hillson, N.J. (2014) PR-PR Cross-Platform Laboratory Automation System. ACS Synthetic Biology 3 (8), 515–524).

Figure S11. Yeast promoter library transformation and assessment

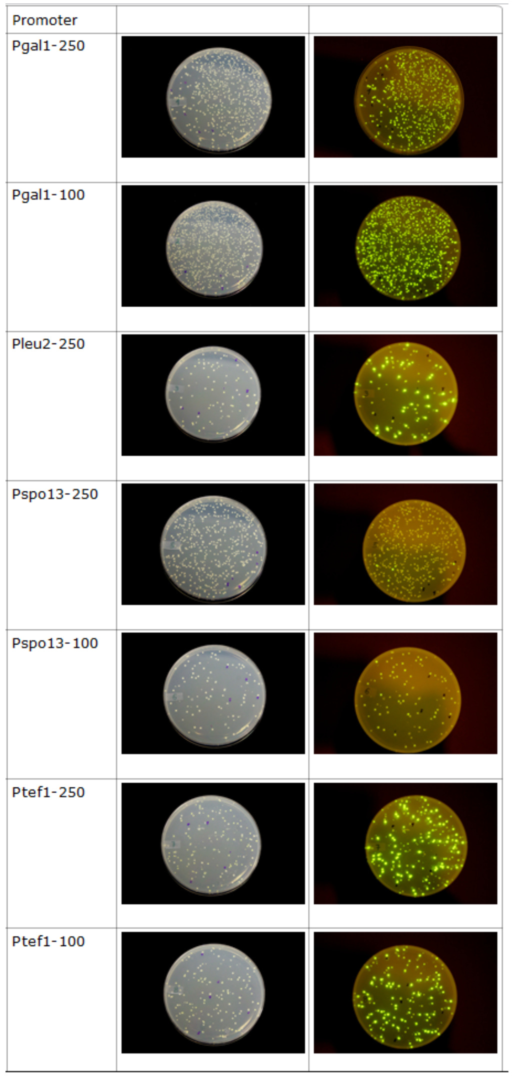

Supplementary Figure 11: Yeast promoter library transformation on-chip and assessment off-chip. First column: promoter name; second column: bright field image of the transformed colonies on the plate; third column: fluorescence image of the colonies. Different promoters regulate different levels of *gfp* expression.

Figure S12. Screen for GFP-positive clones following pETBLUE-GFP transformation into *E. coli*

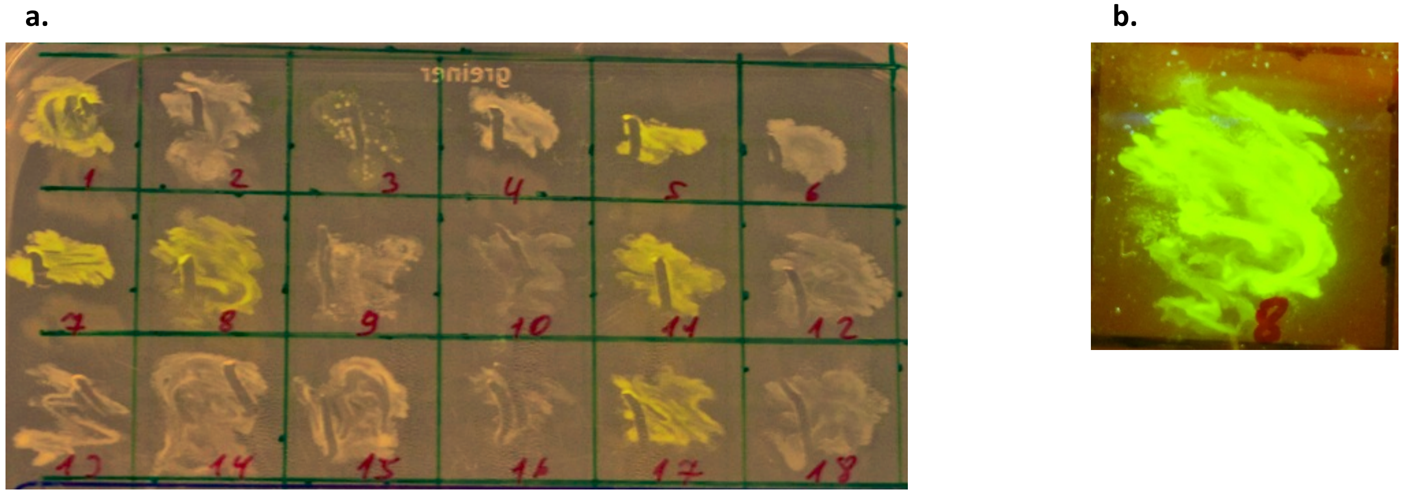

Supplementary Figure 12: Screen for GFP-positive clones following pETBLUE-GFP transformation into *E. coli*. (a) off-chip screening of 18 clones. **(b)** Fluorescence image of GFP-positive colony number 8.

Figure S13. Induction of GFP expression on-chip

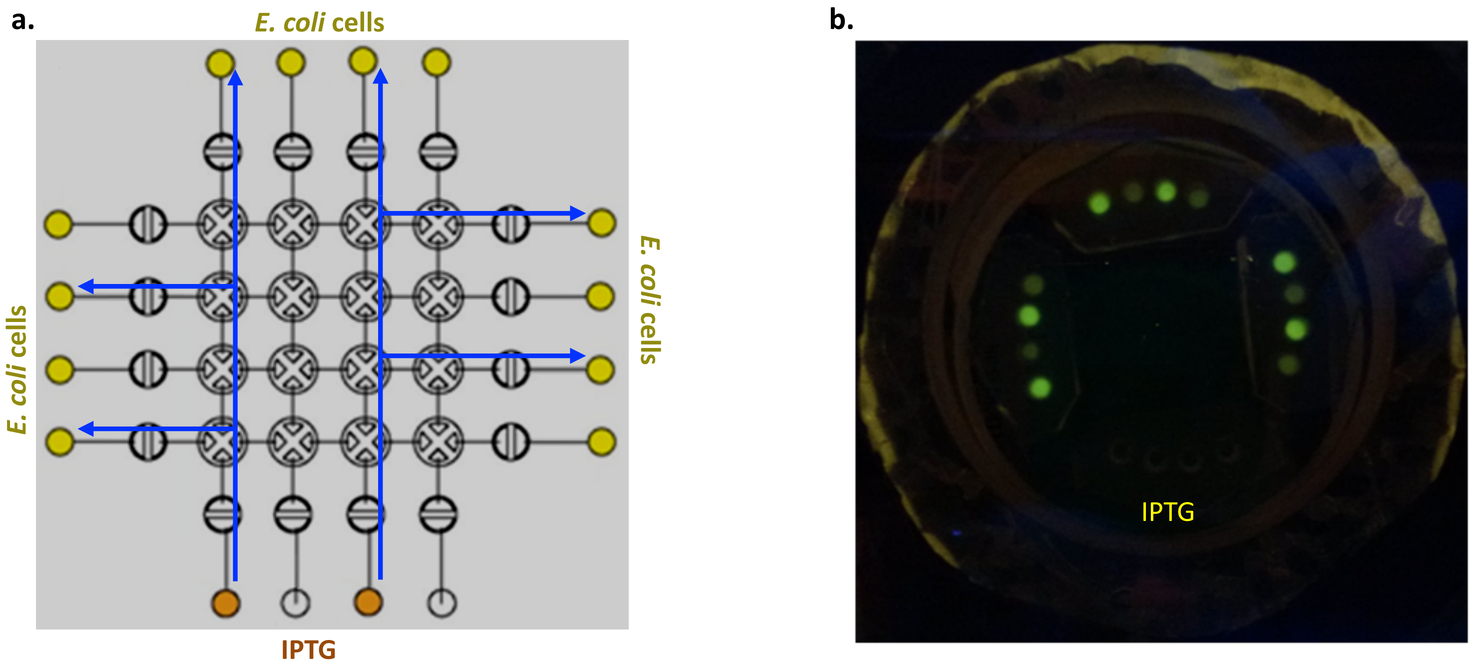

Supplementary Figure 13: Induction of GFP expression on chip. (a) Schematic of induction of GFP expression on-chip. **(b)** Fluorescence image of the chip after induction. The *E. coli* strain containing the constructed plasmid was loaded on-chip into the input wells, and 6 out of the 12 wells were induced with IPTG for *gfp* expression.

Figure S14. MBTH assay

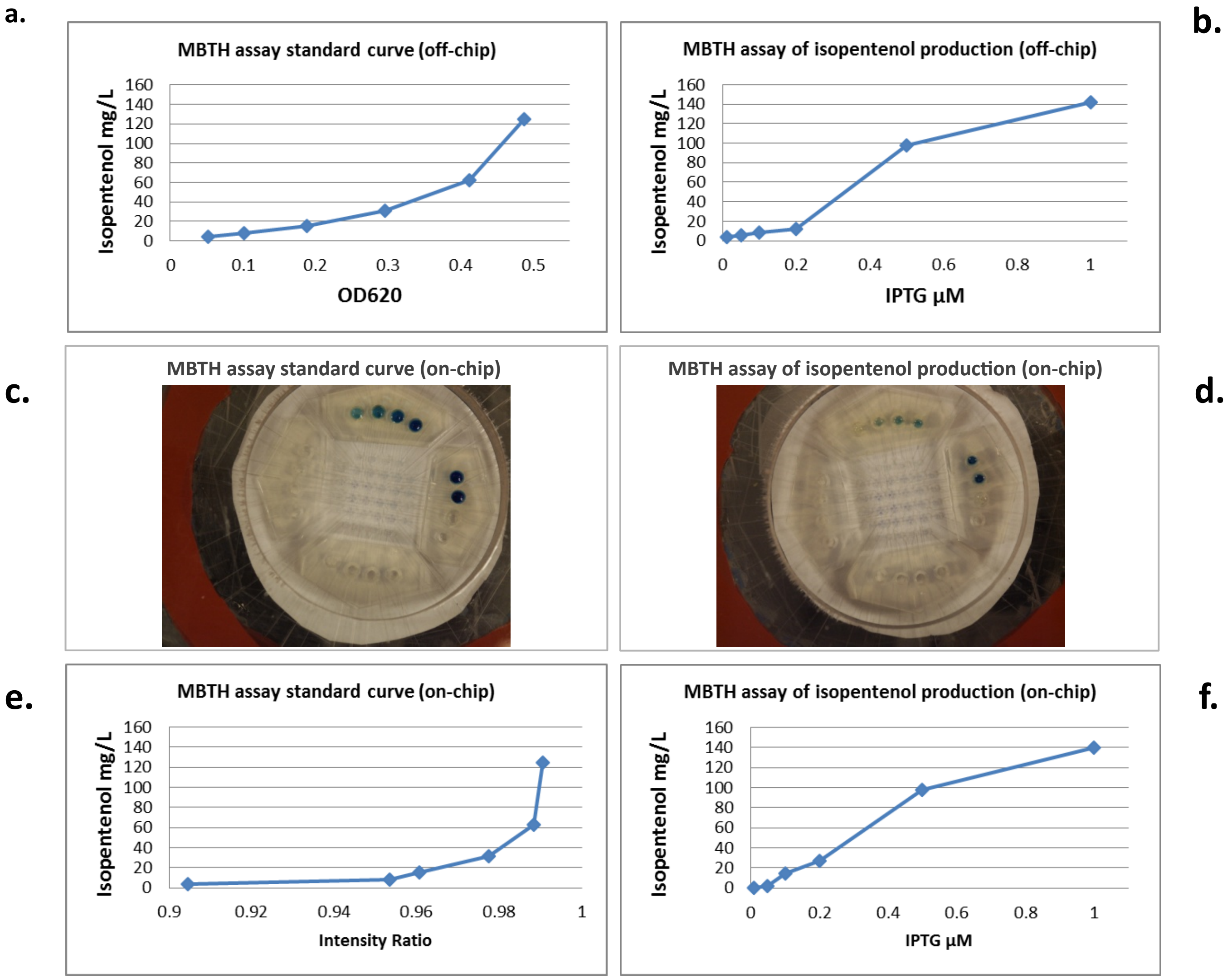

Supplementary Figure 14: MBTH assay. (a) MBTH assay standard curve measured using a plate reader. **(b)** Isopentenol concentration measured by MBTH assay using a plate reader. **(c)** Picture of the on-chip MBTH assay standard curve. **(d)** Picture of the on-chip MBTH assay of isopentenol concentration. **(e)** MBTH assay standard curve measured using on-chip image analysis. **(f)** Isopentenol concentration measured by MBTH assay using on-chip image analysis.

Figure S15. Demonstration of a complete process imparting a desired function into *E. coli*

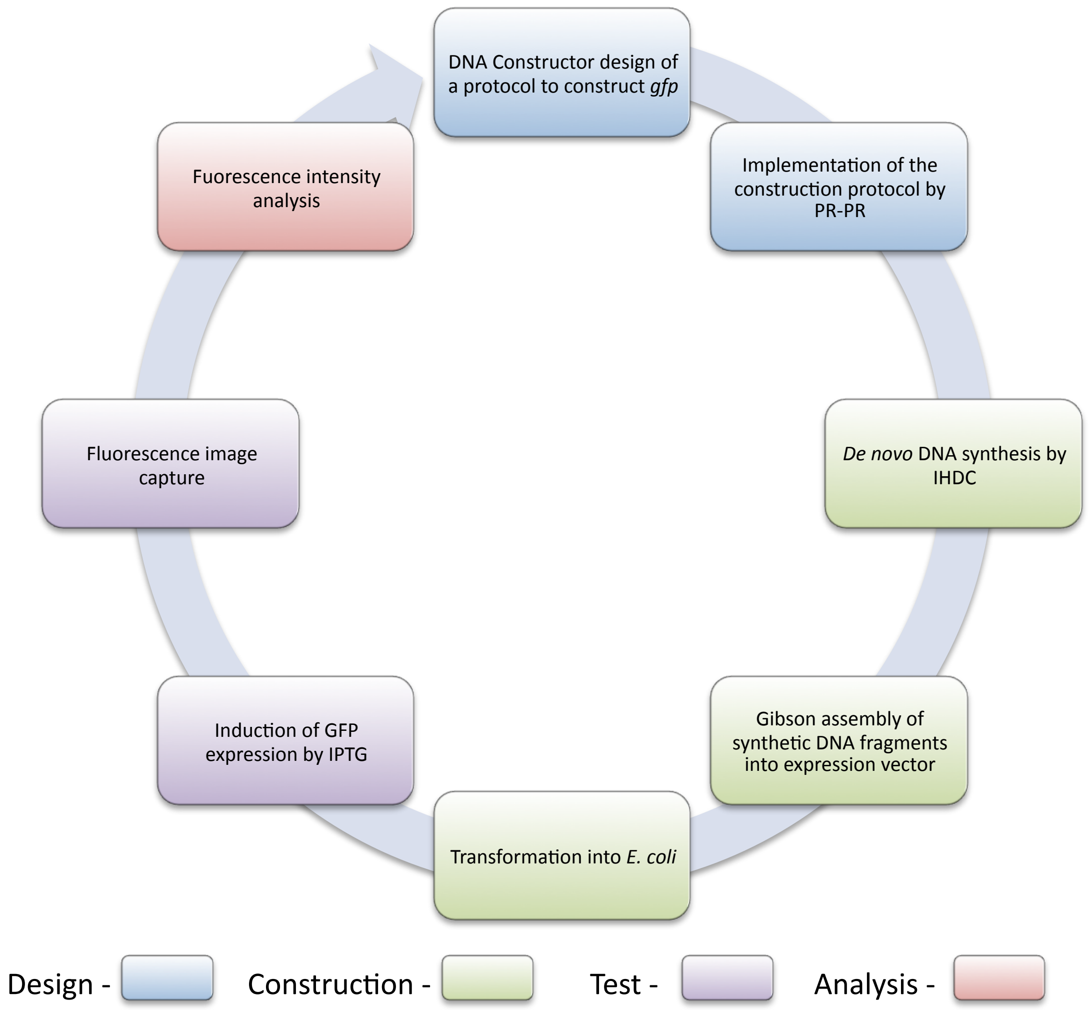

Supplementary Figure 15: Demonstration of a complete process imparting a desired function into *E. coli* - from design to analysis.
